# TLR5 agonists enhance anti-tumor immunity and overcome resistance to immune checkpoint therapy

**DOI:** 10.1038/s42003-022-04403-8

**Published:** 2023-01-12

**Authors:** Caleb Gonzalez, Sarah Williamson, Seth T. Gammon, Sarah Glazer, Joon Haeng Rhee, David Piwnica-Worms

**Affiliations:** 1 Department of Cancer Systems Imaging, University of Texas MD Anderson Cancer Center, Houston, TX 77030 USA; 2 Chonnam National University Medical School, Gwangju, South Korea

**Keywords:** Cancer immunotherapy, Cancer therapeutic resistance, Breast cancer, Chemokines, Immunoediting

## Abstract

Primary and adaptive resistance to immune checkpoint therapies (ICT) represent a considerable obstacle to achieving enhanced overall survival. Innate immune activators have been actively pursued for their antitumor potential. Herein we report that a syngeneic 4T1 mammary carcinoma murine model for established highly-refractory triple negative breast cancer showed enhanced survival when treated intra-tumorally with either the TLR5 agonist flagellin or CBLB502, a flagellin derivative, in combination with antibodies targeting CTLA-4 and PD-1. Long-term survivor mice showed immunologic memory upon tumor re-challenge and a distinctive immune activating cytokine profile that engaged both innate and adaptive immunity. Low serum levels of G-CSF and CXCL5 (as well as high IL-15) were candidate predictive biomarkers correlating with enhanced survival. CBLB502-induced enhancement of ICT was also observed in poorly immunogenic B16-F10 melanoma tumors. Combination immune checkpoint therapy plus TLR5 agonists may offer a new therapeutic strategy to treat ICT-refractory solid tumors.

## Abstract (web summary)

TLR5 agonists in combination with immune checkpoint therapies (ICT) enhance survival in ICT-refractory murine 4T1 mammary carcinoma and B16-F10 melanoma tumors. Low serum levels of G-CSF and CXCL5 correlate with enhanced survival.

## Introduction

Immune checkpoint therapies (ICT) have opened new therapeutic venues against cancer with lasting curative effects^1,2^. Two well-characterized immune checkpoint regulators targeted in ICT are PD-1 and CTLA-4^3,4^. PD-1 modulates immune activity by inhibiting apoptosis of immune-suppressive T-regs, and promoting apoptosis of antigen-specific T cells^5^. PD-1 is also known to inhibit the activation of B-cells^6^. By contrast, CTLA-4 is thought to interfere with T-cell activation^4,5^. However, the majority of cancer patients do not respond to these treatments or relapse after a period of response^7^. A common theme prevalent in the mechanisms of resistance is the failure to elicit a lasting adaptive response due to deficiencies in any one of a number of steps in the tumor antigen presentation process. It is thought that these deficiencies are likely caused by inherent or acquired low antigenic protein expression and/or uncoupling of antigen-presenting signaling in tumor cells or tumor microenvironment and immune activation. Independent of the mechanisms of resistance, it is possible that direct activation of innate immunity could trigger an immune response capable of overcoming tumor resistance to immune checkpoint-based therapies^8^.

Recent evidence indicates that therapies that harness innate immunity show promising antitumor potential^9^. In a number of studies, *Salmonella typhimurium*, a flagellated facultative intracellular bacterium, induces tumor regression in pre-clinical models^10–17^. Building on this concept, therapeutic trials with *Salmonella* species are underway (https://ClinicalTrials.gov/show/NCT03762291). The therapeutic effects of *Salmonella* are likely driven by bacteria antigenicity and activation of host immune-mediated recognition of pathogen-associated molecular patterns by Toll-like receptors (TLRs)^14,18–20^. Not surprising, many TLRs agonist have been shown to elicit antitumor activity^21–24^. In particular, treatment with bacterial flagellin, a TLR5 agonist^25^, results in potent antitumor responses in various xenograft models for colon, breast, and prostate cancer as well as a number of mouse spontaneous tumor models^23,26–31^. Interestingly, higher *Tlr5* expression levels correlate with enhanced survival in breast, lung, and ovarian cancer patients^32^. Although the precise mechanisms of TLR5-mediated antitumor effects remain to be elucidated, it is known that TLR5 mediates innate immune responses against bacterial flagellin^25^, likely through activation of pro-inflammatory pathways, including NF-κB^26,32,33^. Of interest, TLR5 may also mediate adaptive immune activation by acting as a co-stimulatory receptor on CD4^+^ T cells in a way analogous to CD28 co-stimulatory role during naïve CD4^+^ T cell activation^34^. Thus, it is possible that the antitumor responses are a collateral effect of host immune response to flagellin. TLR5-mediated immunogenic response has led to the exploration of flagellin-derived reagents suitable for clinical application. CBLB502 (Entolimod) is a recombinant flagellin protein fragment derived from *Salmonella enterica*, which acts as a TLR5 agonist and activator of the NF-κB inflammatory response^35,36^. In a number of pre-clinical studies, treatment with CBLB502 showed antitumor and anti-metastatic effects through activation of components of the innate immune system^37–41^. Safe systemic administration of CBLB502 has been demonstrated in rodents, non-human primates and humans^35,42,43^ (https://ClinicalTrials.gov/show/NCT01527136) a potentially significant advance compared to treatment with agonists of other TLR family members in the context of cancer therapy^44–46^ (https://ClinicalTrials.gov/show/NCT00960752). Herein we explored combinations of TLR5 agonists and immune checkpoint therapy (ICT) using the immunogenic 4T1 breast cancer solid tumor model and the poorly immunogenic B16-F10 melanoma tumor model, both highly aggressive cancers and refractory to standard therapies^47–49^. Any demonstration of long-term survivors in these models would be considered an advance worthy of further translational efforts.

## Results

### NF-κB activation of 4T1 cells in vitro

**Fig. 1:**
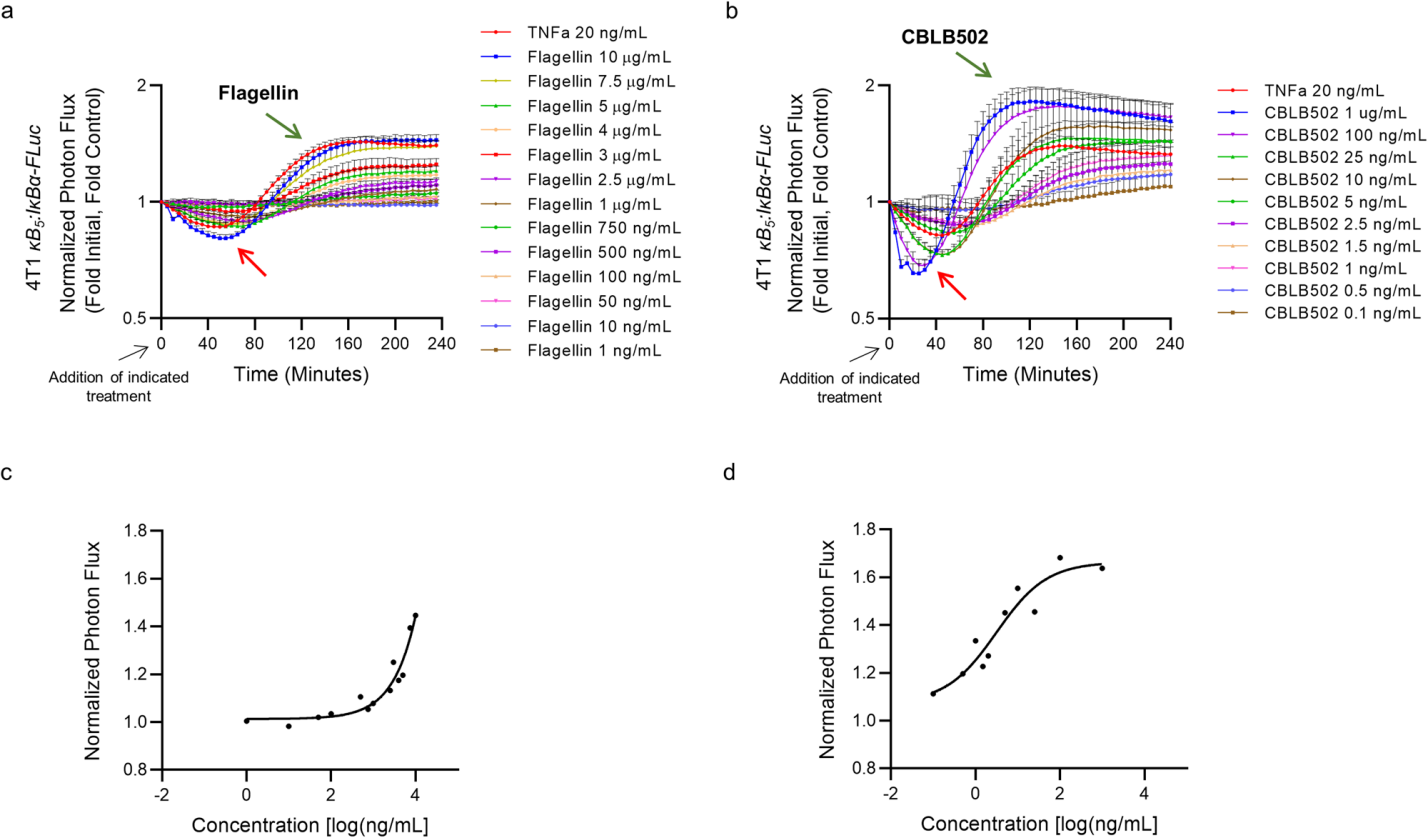
Characterization in vitro of 4T1 murine mammary carcinoma cells response to incubation with TLR5 agonists. 4T1 cells stably expressing *pκB*_*5*_*:IκBαFLuc* were stimulated with the indicated ligand at *t* = 0 and bioluminescence activity imaged every 5 min for 4 h. Data are displayed as normalized photon flux values (average fold-initial, fold-vehicle). **a** 4T1 cells stably expressing *pκB*_*5*_*:IκBαFLuc* were treated with increasing flagellin concentrations: 1 ng/ml (*n* = 7), 10 ng/ml (*n* = 2), 50 ng/ml (*n* = 2), 100 ng/ml (*n* = 7), 500 ng/ml (*n* = 7), 750 ng/ml (*n* = 2), 1 μg/ml (*n* = 7), 2.5 μg/ml (*n* = 7), 3 μg/ml (*n* = 5), 4 μg/ml (*n* = 5), 5 μg/ml (*n* = 7), 7.5 μg/ml (*n* = 5), 10 μg/ml (*n* = 7), and TNFα 20 ng/ml (*n* = 7). Error bars represent S.E.M. for the indicated number of independent experiments. **b** 4T1 cells stably expressing *pκB*_*5*_*:IκBαFLuc* were treated with increasing CBLB502 concentrations: 0.1 ng/ml (*n* = 3), 0.5 ng/ml (*n* = 3), 1 ng/ml (*n* = 3) 1.5 ng/ml (*n* = 3), 2.5 ng/ml (*n* = 3), 5 ng/ml (*n* = 3), 10 ng/ml (*n* = 3), 25 ng/ml (*n* = 3), 100 ng/ml (*n* = 3), 1 μg/ml (*n* = 3), and TNFα 20 ng/ml (*n* = 3). Error bars represent S.E.M. for the indicated number of independent experiments. **c** The half maximal effective concentration (EC50) of flagellin in 4T1 cells is >10^4^ ng/mL in this model. **d** The half maximal effective concentration (EC50) of CBLB502 in 4T1 cells is approximately 3.1 ng/mL in this model.

To evaluate flagella- and CBLB502-mediated NF-κB activation of 4T1 mammary carcinoma cells, we stably transfected 4T1 cells with a *κB5:IκBɑ-FLuc* transcriptional reporter comprised of a concatenated *κB5* promoter region, followed by the bioluminescent *IκBɑ-FLuc* fusion reporter gene^50,51^. This reporter provides a readout of endogenous ligand-induced IκBɑ degradation and production of new IκBɑ-FLuc fusion protein^51^, producing a dynamic two-phase signal. In the cytoplasm, IκBɑ sequesters and inactivates NF-κB dimers. The binding of flagellin (or CBLB502) to TLR5 on the cell surface initiates IKK-mediated kinase activity, and the subsequent phosphorylation, ubiquitination, and targeting for proteasomal degradation of endogenous IκBɑ as well as the reporter fusion protein^32,51^. As expected by this mechanism, this resulted in a reduction of bioluminescent activity during the first 100 min in flagellin-treated cultures (Fig. 1a, red arrow) and the first 80 min in CBLB502-treated cultures (Fig. 1b, red arrow). Subsequently, the released NF-κB dimers translocate to the nucleus and bind to the *κB5* promoter region of the reporter, initiating transcription and translation of new bioluminescent fusion proteins. This resulted in a second phase increase in bioluminescent signal (Fig. 1a, b, green arrows), which after a sufficient period of time, returned to a homeostatic state as previously observed with other NF-κB activating ligands^32,52^. Overall, incubation of 4T1 cells with either flagellin or CBLB502 resulted in a concentration-dependent degradation and subsequent resynthesis of the IκBα-FLuc fusion reporter reflecting the cycle of NF-κB signaling (Fig. 1a, b). The half-maximal effective concentration (EC_50_) for flagellin and CBLB502 were >10^4^ ng/mL and 3.1 ng/mL, respectively, in this cell line, directly demonstrating the enhanced potency of CBLB502 for activating the NF-κB signaling pathway (Fig. 1c, d).

### Cytokine profile of 4T1 cells in vitro

CBLB502 activation of NF-κB pro-inflammatory signaling is mediated through TLR5^35^, a known activator of the innate immune system^25^. Given that CBLB502 is a potent activator of the NF-κB signaling in 4T1 carcinoma cells (Fig. 1b, d), we tested whether CBLB502 was sufficient to elicit stimulatory changes in the 4T1 cell cytokine profile. We measured protein arrays of 62 mouse cytokines secreted into conditioned media from 4T1 reporter cells in response to overnight treatment with CBLB502 (1 µg/mL). Supplementary Fig. 1a identified many upregulated cytokines that activate innate immunity (in order of fold over control): SCF (97-fold), L-selecting (29-fold), CCL11 (7-fold), IL-3 and its receptor IL-3RB (4- and 7-fold respectively), CCL9 (7-fold), CCL20 (6-fold), (IL-12 p40/p70 (5-fold), CCL2 (5-fold), Leptin-R (4-fold), CCL3 (4-fold), IGFBP-5 (3-fold), CCL19 (3-fold), and TNF-α (2-fold), while some exert broader immune regulatory functions, such as VEGF (3-fold), G-CSF (2-fold), and IL-2 (3-fold) (Supplementary Table 1). CXCL13 (0.3-fold), CXCL12 (0.3-fold), IL-6 (0.3-fold), CD40 (0.4-fold), and IL-4 (0.6-fold) showed decreased levels (Supplementary Fig. 1a and Supplementary Table 1). Among these cytokines, CXCL1, CXCL5, and CCL2 showed a statistical detectable increase compared to the vehicle control (two tailed, *p* ≤ 0.05) (Supplementary Fig. 1b). It is noteworthy that these cytokines are known to be chemoattractants for components of innate immunity such as neutrophils and monocytes^53–56^. Overall, incubation of CBLB502 (1 µg/mL) re-programed the cell cytokine signaling profile by upregulating many pro-inflammatory and innate immunity recruiting cytokines.

### Treatment of ICT-refractory 4T1 tumors in vivo

Next, we investigated whether administration of flagellin or CBLB502 could elicit antitumor responses in a syngeneic triple negative breast cancer 4T1 tumor model in vivo. Mammary cell carcinomas were generated in BALB/c mice (5–6 weeks old) by orthotopic injection of 4T1 *FUGW-FL* tumor cells into the right fourth mammary fat pad. Tumor progression of each mouse was assessed weekly using bioluminescence imaging (BLI) and caliper measurements of tumor volume (Supplementary Fig. 2a–h). Bioluminescent signal was detected one-week post orthotopic injection, confirming successful tumor implantation. Tumors were palpable and displayed strong bioluminescence signal two weeks post orthotopic injection, indicating robust tumor growth. Mice were then randomized into four different treatment controls: vehicle control, ICT (anti-PD-1 and anti-CTLA-4), flagellin or CBLB502 treatment, and flagellin or CBLB502 in combination with ICT treatment at the indicated dose (Table 1) and delivery method (Supplementary Table 2).

**Table 1:** Treatment doses and delivery routes.

Treatment	Initial dose and delivery site	Subsequent dose and delivery site
Flagellin	10 μg i.t. or i.p.	2 μg (8×) i.t. or i.p.
CBLB502 High Dose	10 μg i.t. or i.p.	2 μg (8×) i.t. or i.p.
CBLB502 Low Dose	1 μg i.t. or i.p.	0.2 μg (8×) i.t. or i.p.
anti-CTLA-4 (9D9)	200 μg i.p.	100 μg i.p. (3×)
anti-PD-1 (RPM1-14)	200 μg i.p.	100 μg i.p. (3×)

### Flagellin treatment

**Fig. 2:**
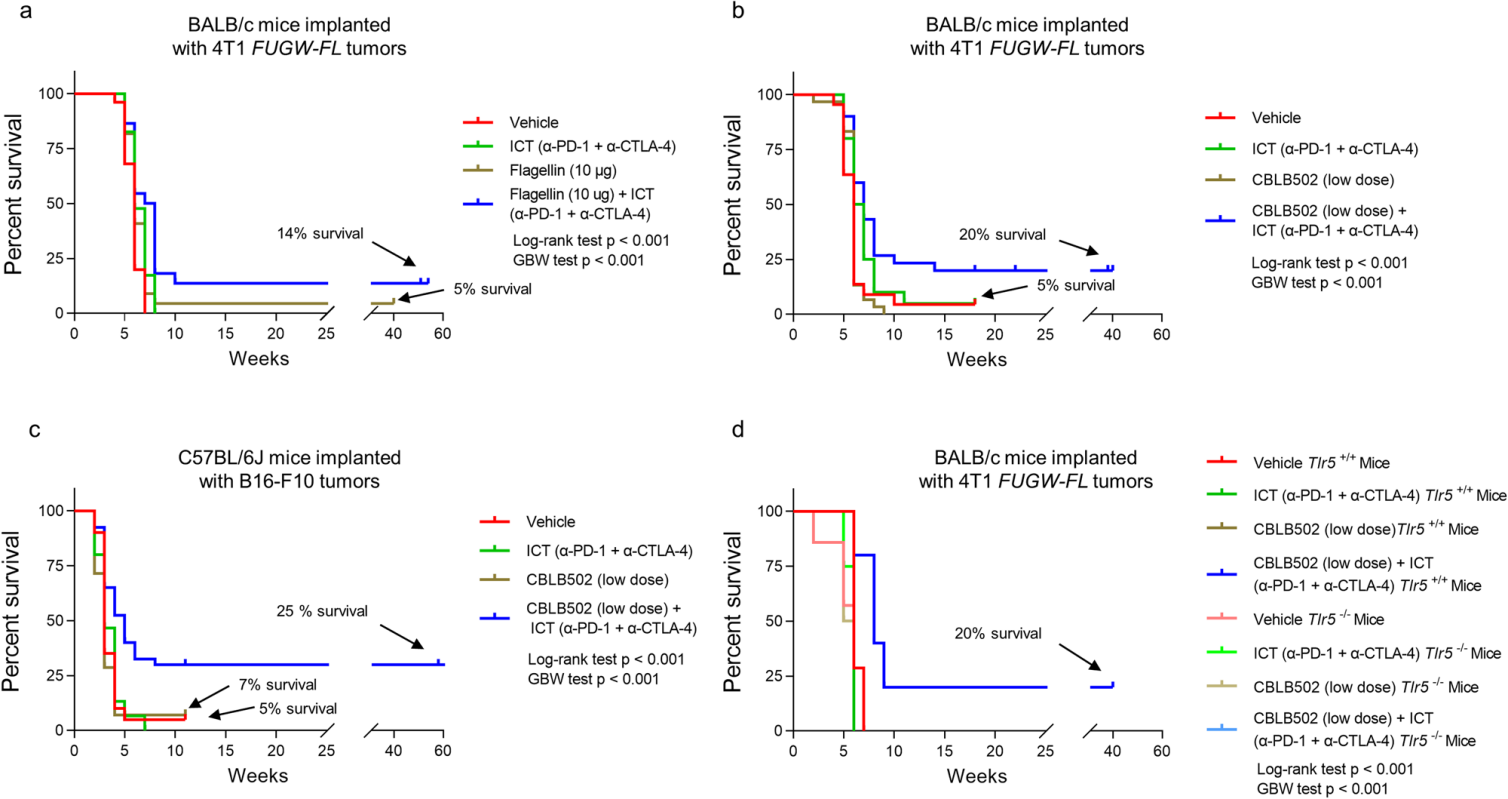
Combination treatment with TLR5 agonist and ICT enhance survival in ICT-refractory tumors and TLR5 host receptors are necessary to elicit the anti-tumor response in a 4T1 tumor model. **a** Kaplan–Meier survival analysis of BALB/c mice implanted with orthotopic 4T1 *FUGW-FL* florescent and bioluminescent reporter cells at week 0 and treated with flagellin with or without ICT from week 2 to week 4. Mice treated with vehicle control (PBS) (*n* = 25), ICT (*n* = 23), flagellin (*n* = 22) and flagellin + ICT treatment (*n* = 22) were compared. Treatment with flagellin + ICT treatment had a statistically significant effect on survival (*p* = 0.0002, Log-rank test; *p* = 0.003, Gehan–Breslow-Wilcoxon test) compared to treatment with vehicle control. Treatment with only ICT also showed statistically significant effect on survival (*p* = 0.01, Log-rank test; *p* = 0.03, Gehan–Breslow–Wilcoxon test) compared to treatment with vehicle control. However, all ICT-treated mice were dead by week eight. Treatment with only flagellin did not show detectable difference (*p* = 0.06, Log-rank test; *p* = 0.08, Gehan–Breslow–Wilcoxon test) compared to treatment with vehicle control. (b) Combination treatment with CBLB502 (low dose) and ICT enhance survival. Kaplan–Meier survival analysis of BALB/c mice implanted with orthotopic 4T1 *FUGW-FL* florescent and bioluminescent reporter tumor cells at week 0 and treated with CBLB502 (low dose) with or without ICT from week 2 to week 4. Mice treated with vehicle control (PBS) (*n* = 22), ICT (*n* = 25), CBLB502 (low dose) (*n* = 30), and CBLB502 (low dose) + ICT (*n* = 30) were compared. Treatment CBLB502 (low dose) + ICT had a statistically detectable effect on survival (*p* = 0.002, Log-rank test; *p* = 0.001, Gehan-Breslow-Wilcoxon test) compared to treatment with vehicle control. ICT alone treatment did not show a detectable statistical difference by Log-rank test (*p* = 0.1), but did show a detectable statistical difference with the Gehan–Breslow–Wilcoxon test (*p* = 0.04). CBLB502 (low dose) alone treatment did not show detectable statistical difference (*p* = 0.8, Log-rank test; *p* = 0.3 Gehan–Breslow–Wilcoxon test). **c** Kaplan–Meier survival analysis of C57BL/6J mice implanted with B16-F10 cells at Day 0 and treated with CBLB502 with or without ICT three days post tumor implantation. Mice treated with vehicle control (PBS) (*n* = 20), ICT (n = 15), CBLB502 (*n* = 14) and CBLB502 + ICT treatment (*n* = 39) were compared. Treatment with CBLB502 + ICT treatment had a statistically significant effect on survival (*p* = 0.003, Log-rank test; *p* = 0.01, Gehan–Breslow–Wilcoxon test) compared to treatment with vehicle control. **d** Kaplan–Meier survival analysis of BALB/c *Tlr5*^+/+^ or *Tlr5*^−/−^ mice implanted with orthotopic 4T1 *FUGW-FL* florescent and bioluminescent reporter tumor cells at week 0 and treated with vehicle control (PBS) (*n* = 7, for each vehicle cohort), ICT (*n* = 2 and *n* = 4 for *Tlr5*^+/+^ and *Tlr5*^-/-^ mice respectively), CBLB502 (*n* = 2 and *n* = 5 for *Tlr5*^+/+^ and *Tlr5*^−^^/−^ mice respectively), and CBLB502+ICT treatment (*n* = 10, for each CBLB502 + ICT cohort) were compared. *Tlr5*^+/+^ mice treated with CBLB502 (low dose) + ICT had a statistically detectable effect on survival when compared to *Tlr5*^+/+^ mice treated with vehicle control (*p* < 0.001, Log-rank test; *p* < 0.01, Gehan−Breslow−Wilcoxon test) or *Tlr5*^−^^/−^ mice treated with CBLB502 (i.t.) + ICT (*p* < 0.001, Log-rank test; *p* < 0.001, Gehan−Breslow−Wilcoxon test). *Tlr5*^+/+^ mice treated with vehicle control showed a detectable statistical difference in survival when compared to *Tlr5*^−^^/−^ mice treated with vehicle control (*p* < 0.03, Log-rank test; *p* < 0.03, Gehan−Breslow−Wilcoxon test).

The overall survival combined from four independent experiments is shown in Fig. 2a. All mice under vehicle control (*n* = 25) or ICT only treatment (*n* = 23) died by the end of the study, confirming the ICT-refractory status of the 4T1 breast cancer model. Of note, one vehicle-treated mouse was identified as an outlier using the ROUT Method for having low photon flux measurement at week 2 before treatment began and was omitted from the study. Tumor-free mice were observed in intratumoral flagellin only treatment (10 µg/mouse initial dose) (*n* = 22) (one survivor, *p* = 0.06, Log-rank test) and in intratumoral flagellin + ICT treatment (*n* = 22) (three survivors, *p* = 0.001, Log-rank test) (Fig. 2a). Mice under vehicle control and flagellin only treatment controls showed steady bioluminescent signal with increases in tumor volumes (Supplementary Fig. 2a, c). Interestingly, ICT-treated mice (ICT only or flagellin + ICT-treated mice) showed a sharp decreased in bioluminescent signaling one-week post initiation of treatment (Supplementary Fig. 2b, d, left panel); however, the decrease in bioluminescent signaling was not accompanied by an overall decrease in tumor volume (Supplementary Fig. 2b, d, right panel). At least two possibilities could account for this observation: tumor cell death accompanied by an increased in cellular immune infiltrate or selective loss or silencing of the bioluminescent cassette. Though not mutually exclusive, these results overall pointed to an ICT-induced remodeling in the tumor microenvironment with abundant immune cell infiltrates, which was not predictive of survival per se.

### CBLB502 treatment

Given that CBLB502 showed greater potency and efficacy for activation of NF-κB signaling than flagellin (Fig. 1b, d) and that CBLB502 stimulated/recruited innate immune activating cytokines (Supplementary Fig. 1a, b, and Supplementary Table 1), we tested whether intratumoral treatment with CBLB502 (low dose) could prompt a stronger antitumor response than intratumoral treatment with flagellin. The overall survival combined from four independent experiments (Supplementary Table 2) is shown in Fig. 2b. One mouse from the vehicle control group (*n* = 22) was unexpectedly tumor-free by the end of the experiment. This mouse showed comparable bioluminescent signal during the first two weeks post tumor implantation, indicating that sufficient cells were implanted for tumor growth (Supplementary Fig. 2e, mouse 20). However, it is noteworthy that no palpable tumor was detected during the course of the experiment making it likely that the mouse sequestered the tumor cells before the tumor could establish significant growth. Treatment with CBLB502 alone (low dose) (*n* = 30) resulted in steady bioluminescent signal and tumor growth with no survivors (Supplementary Fig. 2g). ICT treatment alone (*n* = 25) resulted in one tumor-free mouse (Supplementary Fig. 2f, mouse 23) and one mouse that at first seemed to respond to treatment, but later slowly developed a tumor (Supplementary Fig. 2f, mouse 18). Consistent with previous results, ICT-treated mice (ICT only or CBLB502 (low dose) + ICT treatments) showed a sharp decrease in bioluminescent signal one-week post treatment initiation (Supplementary Fig. 2f, h), consistent with a significant initial loss of 4T1 tumor cells. Most importantly, CBLB502 (low dose) + ICT treatment resulted in 20% tumor-free mice (*n* = 30) (*p* = 0.001, Log-rank test; *p* = 0.001, Gehan−Breslow−Wilcoxon test) (Fig. 2b).

We tested a higher intratumoral CBLB502 dose (CBLB502 high dose), which is comparable to the dose administered to mice in the flagellin treatment cohort (Table 1). In three independent experiments (Supplementary Table 2), treatment with CBLB502 (high dose) resulted in one tumor free mouse in the CBLB502 (high dose) only treatment (*n* = 15) with no survivors in any of the other treatments (vehicle (*n* = 19), ICT (*n* = 17), CBLB502 (high dose) + ICT (n = 15)) (Supplementary Fig. 3a–e). Interestingly, one additional mouse in the CBLB502 (high dose) only treatment showed delayed tumor growth (Supplementary Fig. 3d, mouse 3). The lone-survivor (Supplementary Fig. 3d, mouse 5) showed a statistically detectable difference in survival from its vehicle control (*p* = 0.01, Log-rank test; *p* = 0.01, Gehan–Breslow–Wilcoxon test).

We further explored whether systemic delivery of CBLB502 (low dose) via intraperitoneal (i.p.) injections could elicit a similar response to intra-tumoral delivery of CBLB502 (low dose). In two independent experiments (Supplementary Fig. 4a–e, Supplementary Table 2), CBLB502 (low dose) i.p. + ICT treatment resulted in 10% long-term survivors (*n* = 20) (*p* = 0.01, Log-rank test; *p* = 0.01, Gehan–Breslow–Wilcoxon test) (Supplementary Fig. 4e, mice 1 and 5). Whereas vehicle control (*n* = 8) and CBLB502 alone (low dose, i.p. delivery, *n* = 20) resulted in no long-term survivors (Supplementary Fig. 4b, d), ICT-treated mice (*n* = 6) resulted in one long-term survivor in this experiment (*p* = 0.4 and Log-rank test; *p* = 0.3 Gehan–Breslow–Wilcoxon test, compared to vehicle control) (Supplementary Fig. 4c, mouse 3).

### Treatment of ICT-refractory B16-F10 melanoma tumors in vivo

We further investigated whether combination treatment of CBLB502 and ICT could also elicit antitumor responses in a poorly immunogenic tumor, such as the B16-F10 melanoma tumor model. Melanoma tumors were generated in C57BL/6J (6–9 weeks old) by subcutaneous injection of B16-F10 tumor cells into the right dorsal flank. Three days after tumor implantation, mice were randomized into four different treatment controls: vehicle control, ICT (anti-PD-1 and anti-CTLA-4), CBLB502 treatment, and CBLB502 in combination with ICT treatment at the indicated dose and delivery method (Supplementary Table 3). The overall survival from four independent experiments is shown in Fig. 2c. Tumor progression for each mouse was assessed bi-weekly using caliper measurements of tumor volume (Supplementary Fig. 5). Out of four independent experiments, one vehicle control mouse did not develop a palpable tumor during the duration of the experiment (Supplementary Fig. 5a, mouse 18). All ICT only treatment mice (*n* = 15) died by the end of the study (Supplementary Fig. 5b), confirming the ICT-refractory status of the B16-F10 melanoma tumor model without the addition of a vaccine^57^. Only one mouse in the CBLB502 treatment (*n* = 14), did not develop a tumor by the end of the study (Supplementary Fig. 5c, mouse 14). However, survival curves showed no detectable statistical difference with vehicle control group (*p* = 0.5, Log-rank test; *p* = 0.3, Gehan–Breslow–Wilcoxon test) (Fig. 2c). Combination treatment with CBLB502 and ICT resulted in 25% tumor-free mice (*n* = 39) by week 78 of the study (*p* = 0.001, Log-rank test; *p* = 0.003, Gehan–Breslow–Wilcoxon test) (Fig. 2c and Supplementary Fig. 5d). Thus, combination treatment with TLR5 agonists and ICT enhanced survival in vivo from two independent ICT-refractory tumor models.

### Tlr5 knockout mice

We tested whether TLR5 host receptors are necessary to elicit the anti-tumor response observed in mice treated with CBLB505 in combination with ICT treatments (Fig. 2d)). *Tlr5*^+/+^ and *Tlr5*^–^^/–^ mice were challenged with 4T1 *FUGW-FL* (*Tlr5*^+/+^) and treated as in previous experiments (Supplementary Table 4). Kaplan–Meier survival curve from two independent experiments are shown in Fig. 2d. All mice under vehicle control died by the end of the study (Supplementary Fig. 6a, e). However, it is noteworthy that the survival curve for *Tlr5*^–^^/–^ vehicle control mice (*n* = 7) showed a significant difference to the survival curve of *Tlr5*^+/+^ vehicle control mice (*n* = 7) (*p* = 0.03, Log-rank test; *p* = 0.03, Gehan−Breslow−Wilcoxon test) (Fig. 2d). All ICT- or CBLB502-treated mice in both cohort groups were dead by week 6 (Supplementary Fig. 6b, c, f, and g). Consistent with previous results (Fig. 2b), *Tlr5*^+/+^ mice treated with CBLB502 (low dose) + ICT treatments resulted in 20% tumor-free mice (*n* = 10) (Fig. 2d and Supplementary Fig. 6d, mice 2 and 4). Whereas, all *Tlr5*^–^^/–^ mice treated with CBLB502 (low dose) + ICT treatments died by the end of the study (*n* = 10) (*p* = 0.001, Log-rank test; *p* = 0.01, Gehan–Breslow–Wilcoxon test, compared with *Tlr5*^+/+^ mice treated with CBLB502 (low dose) + ICT treatments) (Fig. 2d and Supplementary Fig. 6h). These results indicate that TLR5 host receptors are necessary for anti-tumor responses observed in mice treated with CBLB502 and ICT treatments.

### Tumor re-challenge experiments and immune memory

**Fig. 3:**
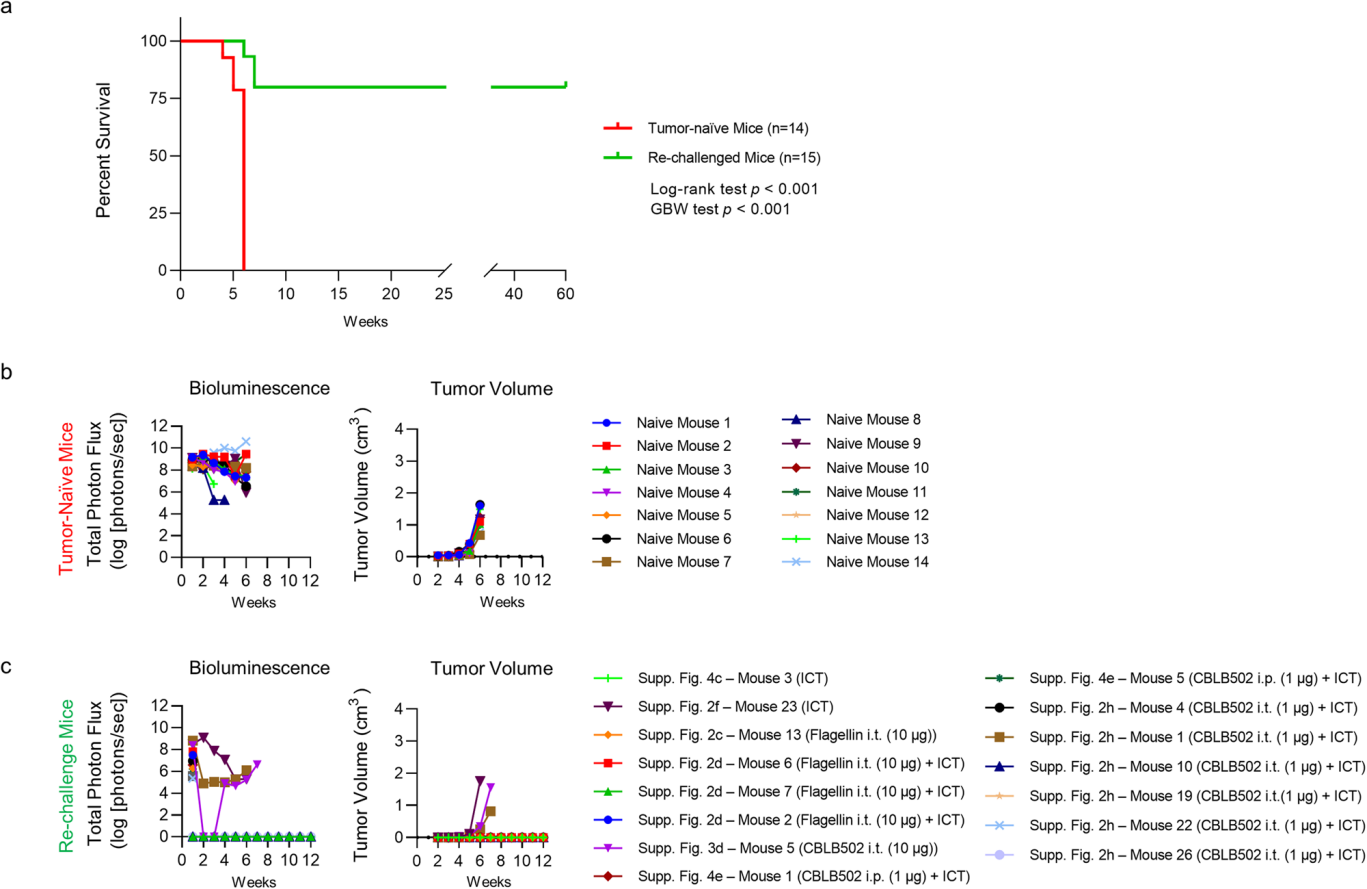
Immune memory in 4T1 *FUGW-FL* tumor-bearing mice. **a** Kaplan–Meier survival analysis of BALB/c mice that survived at least 18 weeks post 4T1 *FUGW-FL* tumor implantation and showed no sign of tumor, but were re-challenged with orthotopic 4T1 *FUGW-FL* tumor cells in the contralateral (left) fourth mammary fat pad (*n* = 15) and age-matched control tumor-naïve mice (*n* = 14). All mice were injected at week 0 with 4T1 *FUGW-FL* tumor cells and tumors were allowed to grow without therapeutic intervention. Re-challenged mice showed 80% survival rate (*p* = 0.0001, Log-rank test; *p* = 0.0001 Gehan–Breslow–Wilcoxon). **b** Tumor size of tumor-naïve mice (*n* = 14) measured by bioluminescence imaging (total photon flux, left panel) and caliper measurements (tumor volume, right panel). All tumor-naïve, tumor challenged-mice died by week 6. **c** Tumor size of re-challenged mice (*n* = 15) measured by bioluminescence imaging (total photon flux, left panel) and caliper measurements (tumor volume, right panel). Only three tumor survivor mice died due to tumor burden: Supplementary Fig. 2f-mouse 23 (ICT), Supplementary Fig. 2h-mouse 1 (CBLB502 1 µg (i.t.) + ICT), and Supplementary Fig. 3d-mouse 5 (CBLB502 10 µg (i.t.)) and, (Supplementary Table 5). All other tumor survivor mice were tumor-free for at least 60 weeks post orthotopic tumor re-challenge.

Mice that showed complete tumor regression were re-challenged by orthotopic injections of 4T1 *FUGW-FL* cells into the opposite (left) fourth mammary fat pad without any additional therapy (Supplementary Table 5). Figure 3a shows overall survival from the re-challenge experiment. All untreated tumor-naïve, tumor-bearing, and aged-matched control mice died by week six, confirming the aggressive potential of the tumor cell cohort, whereas 80% of re-challenged mice were tumor-free for at least 60 weeks post tumor implantation (Fig. 3a–c). One re-challenged mouse was excluded from the survival curve shown in Fig. 3a because the cause of death was unrelated to tumor implantation (Supplementary Fig. 2e – vehicle, mouse 20, died during blood withdrawal for cytokine profiling at week three). Furthermore, one-week post re-challenge, this mouse showed comparable bioluminescent signal to other re-challenge mice (Supplementary Fig. 7), indicating successful tumor implantation. However, no bioluminescent signals or palpable tumor were detected thereafter during the first three weeks of the experiment, indicating that the mouse likely rejected 4T1 *FUGW-FL* tumor implantation. Nonetheless, overall, these results indicated that the observed curative effects were likely due to acquired memory for anti-tumor immunity.

### Peripheral blood cytokine profile in vivo

**Fig. 4:**
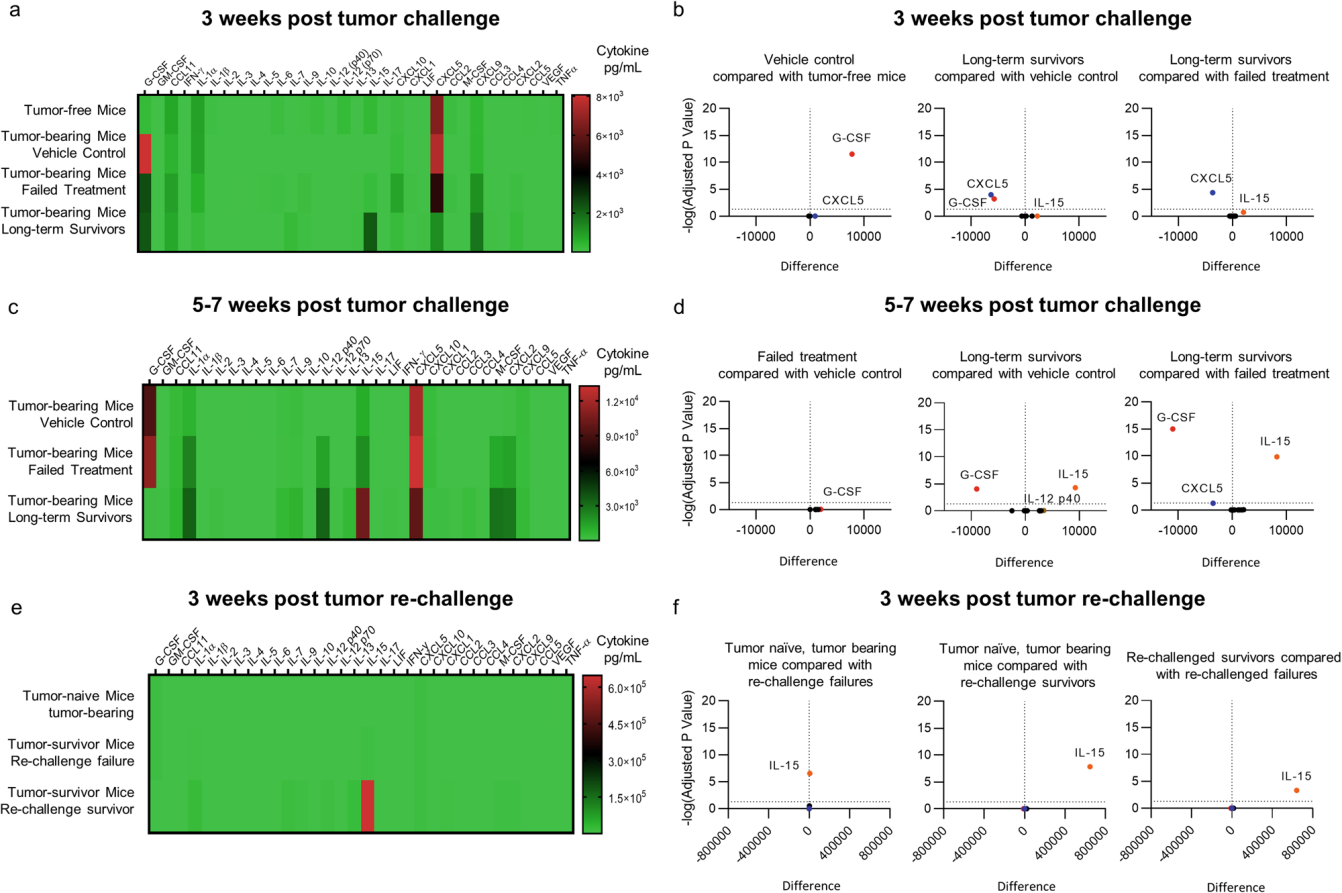
Peripheral blood cytokine profile in vivo. **a** Heatmap of 32 peripheral blood cytokines of tumor-free mice and mice challenged with 4T1 *FUGW-FL* tumors three weeks post orthotopic tumor implantation: tumor-free mice (healthy mice, no tumor implanted); tumor-bearing mice, vehicle (PBS) control (non-survivors), tumor-bearing mice, failed treatment (non-survivors), and tumor-bearing mice, long-term survivors. **b** Volcano plot highlighting detectable statistical difference between peripheral blood cytokines three weeks post orthotopic tumor challenge. **c** Heatmap of 32 peripheral blood cytokines of mice challenged with 4T1 *FUGW-FL* tumors 5 to 7 weeks post tumor implantation: tumor-bearing mice, vehicle (PBS) control (non-survivors) at week 5; tumor-bearing mice, failed treatment (non-survivors), weeks 5 to 7; and tumor-bearing mice, long-term survivors during weeks 6 and 7. **d** Volcano plot highlighting detectable statistical difference between peripheral blood cytokines taken between 5 and 7 weeks post orthotopic tumor challenge. **e** Heatmap of 32 peripheral blood cytokines of mice re-challenged with 4T1 *FUGW-FL* tumor: tumor naïve mice, tumor-bearing (non-survivors), tumor survivor mice, re-challenge failure (non-survivors) and tumor survivor mice, re-challenge survivor (long-term survivors) taken three weeks post orthotopic 4T1 *FUGW-FL* tumor implantation. **f** Volcano plot highlighting detectable statistical difference between peripheral blood-bourne cytokines taken three weeks post orthotopic tumor re-challenge.

To expand on our understanding of the mechanisms of response and characterize changes elicited by ICT and CBLB502 therapies alone or in combination, we assayed 32 peripheral blood-borne cytokines from aged-matched tumor-free mice (healthy mice, no tumor implantation) and 4T1 *FUGW-FL* tumor-bearing mice under our treatment cohorts: tumor-bearing, vehicle control; tumor-bearing, treatment failure; and tumor-bearing, treatment responders (Supplementary Fig. 8a–e), during week 3 (Fig. 4a) and week 10 (Supplementary Fig. 8f) and weeks 5 to 7 (Fig. 4c) for 4T1 *FUGW-FL* tumor-bearing mice cohorts only. Of note, tumor-free healthy mice showed variable expression of cytokines between weeks 3 and 10 of the study, likely reflecting physiological changes in cytokine expression (Fig. 4a and Supplementary Fig. 8f, tumor-free mice).

Four profiles emerged from our analysis. First, it is noteworthy that levels of granulocyte-colony stimulating factor (G-CSF), a cytokine involved in the proliferation and differentiation of granulocytes and neutrophils^58^ and associated with poorer survival in cervical, non-small cell lung cancer, colon, melanoma, and skin cancers^59–63^, showed a statistical increase in tumor-bearing mice, vehicle control compared with tumor-free mice three weeks post 4T1-*FUGW-FL* tumor implantation (Fig. 4a, b, left panel, Supplementary Table 6), and a statistical decrease when compared with tumor-bearing mice, treatment responders (long-term survivors) (Fig. 4a, b, middle panel, Supplementary Table 6). Although there was no statistical difference between tumor-bearing mice that failed treatment (non-survivors), and tumor-bearing mice, treatment responders (long-term survivors) three weeks post 4T1 *FUGW-FL* tumor implantation (Fig. 4a, b, right panel, Supplementary Table 6), there was a statistical decrease during weeks 5 through 7 between these two groups of mice (Fig. 4c, d, right panel, Supplementary Table 7). Consistent with these results, tumor-bearing mice, treatment responders also showed a statistical decrease during weeks 5 through 7 when compared with tumor-bearing mice, vehicle control (Fig. 4c, d, middle panel, Supplementary Table 7). Second, CXCL5, a chemokine involved in recruitment of myeloid derived suppressor cells (MDSCs) to the tumor microenvironment^64–67^ and associated with poor survival in renal, liver, pancreatic, and cervical cancer^68^, showed a statistical decrease in tumor-bearing mice that responded to treatment (long-term survivors) compared with tumor-bearing mice that failed treatment (non-survivors) three weeks post 4T1 *FUGW-FL* tumor implantation (Fig. 4a, b, right panel, Supplementary Table 6). Third, we find that during weeks 5 through 7 post tumor implantation, IL-15, a pro-inflammatory and immune-activating cytokine that promotes survival, differentiation, proliferation, and activation of natural killer (NK), CD8^+^ T cells, and B cells^69–75^, showed a statistical increase in tumor-bearing mice that responded to treatment (long-term survivors) compared with tumor-bearing mice that failed treatment (non-survivors) (Fig. 4c, d, right panel, Supplementary Table 7) or with tumor-bearing mice vehicle control (Fig. 4c, d, middle panel, Supplementary Table 7). By contrast, there was no statistical difference between tumor-bearing mice, vehicle control, compared with tumor-bearing mice that failed treatment (non-survivors) (Fig. 4c, d, left panel, Supplementary Table 7). Fourth, although not statistically significance based on two-way ANOVA tests, tumor-bearing mice that responded to treatment showed an overall increase trend for immune-activating cytokines compared to tumor-bearing mice that failed treatment during weeks 5 through 7 post tumor implantation: GM-CSF (11-fold)^76^, IL-2 (15-fold)^77^, IL-13 (8-fold)^78^, IFN-γ (7-fold)^79^, and CCL3 (four-fold)^80^. Of note, CXCL1, a chemokine associated with tumor immune suppression^81^, showed a 4-fold increase over tumor-bearing mice that failed treatment (Supplementary Table 7). IL-10, a cytokine involved in immune suppression through inhibition of antigen-presenting cells (APC) and the activation of immune suppressive T-reg cells^82^, showed a two-fold decreased in mice that responded to treatment compared with mice that failed treatment (Supplementary Table 7). Interestingly, IL-10 antagonists are currently being explore as anti-tumor immune therapy in combination with TLR agonists and other immunostimulatory treatments^83^. Finally, tumor-bearing mice that responded to treatment (long-term survivors) showed statistical increases in IL-1α, IL-15, CXCL5, and IL-12 p40 when compared to tumor-free control mice, indicating a distinctive immune activating systemic cytokine profile ten weeks post 4T1 *FUGW-FL* tumor implantation (Supplementary Fig. 8f, g).

### Peripheral blood cytokine profile of re-challenged mice

Tumor re-challenged and tumor-naïve mice cytokines were assayed three weeks post 4T1 *FUGW-FL* implantation. Mice that were tumor-free for at least 60 weeks post re-challenge (tumor survivor mice, re-challenge survivor) revealed a distinctive cytokine profile from those mice that were re-challenged, but developed tumors (tumor survivor mice, re-challenge failure) (Fig. 4a). Interestingly, similar to previous results with challenge mice (Fig. 4c, d), IL-15 showed a statistical increase in long-term survivors (tumor survivor mice, re-challenge survivor) compared with mice that were re-challenge, but did not survive (tumor survivor mice, re-challenge failure) (Fig. 4e, f, right panel, Supplementary Table 8) and with tumor naïve, tumor bearing mice that also did not survived (Fig. 4e, f, middle panel, Supplementary Table 8). Interestingly, re-challenge mice that did not survive (tumor survivor mice, re-challenge failure) also showed a statistical increase when compared to tumor naïve mice, tumor bearing (Fig. 4e, f, left panel, Supplementary Table 8), but to a lesser extent than long-term survivors (tumor survivor mice, re-challenge survivor). Moreover, levels of G-CSF also trended lower in long-term survivors (tumor survivor mice, re-challenge survivor) (19-fold decreased) when compared with tumor-naïve, tumor-bearing mice (non-survivors) (Supplementary Table 8), but these values fell shy of statistical significance (two-way ANOVA). Mice that were re-challenged, but did not survive (tumor survivor mice, re-challenge failure), showed similar levels of G-CSF to tumor-naïve, tumor-bearing mice at this time point (Supplementary Table 8). In addition, four profiles emerged from our analysis. First, cytokines that were upregulated in both re-challenge failure mice and re-challenge survivor mice, but with a far greater increase in re-challenge survivors: LIF (5-fold compared with 420-fold), CXCL1 (3-fold compared with 120-fold), IL-2 (7-fold compared with 260-fold), IL-7 (100-fold compared with 3700-fold), IL-12 p70 (10-fold compared with 240-fold), CCL4 (3-fold compared with 58-fold), CCL11 (3-fold compared with 27-fold), CCL3 (3-fold compared with 20-fold), IL-1α (4-fold compared with 22-fold), IL-10 (55-fold compared with 210-fold), IL-1β (3-fold compared with 6-fold), CXCL2 (8-fold compared with 17-fold), IL-4 (21-fold compared with 41-fold), IL-9 (4-fold compared with 7-fold), and M-CSF (40-fold compared with 62-fold) (Supplementary Table 8). Second, cytokines that were downregulated in both groups, but to a greater extent in the re-challenge failure cohort: IL-3 (0.4 compared with 0.7 decrease), IL-5 (0.3 compared with 0.5 decrease), and CXCL10 (0.3 compared with 0.5 decrease) (Supplementary Table 8). Third, cytokines that showed differential regulation between the two cohort: IL-12 p40 (0.4 decrease compared with two-fold increase), TNFα (0.5 decrease compared with a two-fold increase), IL-6 (0.7 decrease compared with two-fold increase) (Supplementary Table 8). Fourth, cytokines that did not change in one population, but did in another: re-challenge survivor mice upregulated IL-13 (53-fold), CXCL9 (12-fold), IFN-γ (9-fold), CCL5 (three-fold), whereas re-challenge failure mice did not upregulate these cytokines greater than two-fold. CXCL5 showed a 0.8 decrease in long-term survivors, but no change in mice that failed to survive. IL-17 showed a two-fold increase in mice that failed to survive, but no change in long-term survivors (Supplementary Table 8). Taken together, these results showed a distinctive adaptive immune-activating cytokine profile in those mice that survived the re-challenge experiment.

### Tumor immune infiltrate

To further elucidate the effects of CBLB502 and ICT therapies alone or in combination, we explored changes to the tumor immune microenvironment in response to these therapies and inquired if changes to immune cells infiltrate correlated with increased likelihood of survival. We used a unique strategy based on bioluminescence signal as a prognostic tool for survival outcomes of mice that were euthanized at three weeks post 4T1 *FUGW-FL* tumor implantation for tumor extraction and subsequent tumor immune infiltrate flow cytometry profiling, in effect at a time before outcomes for individual mice would be known, but bioluminescence signal would be predictive.

### Prognostic tool

**Fig. 5:**
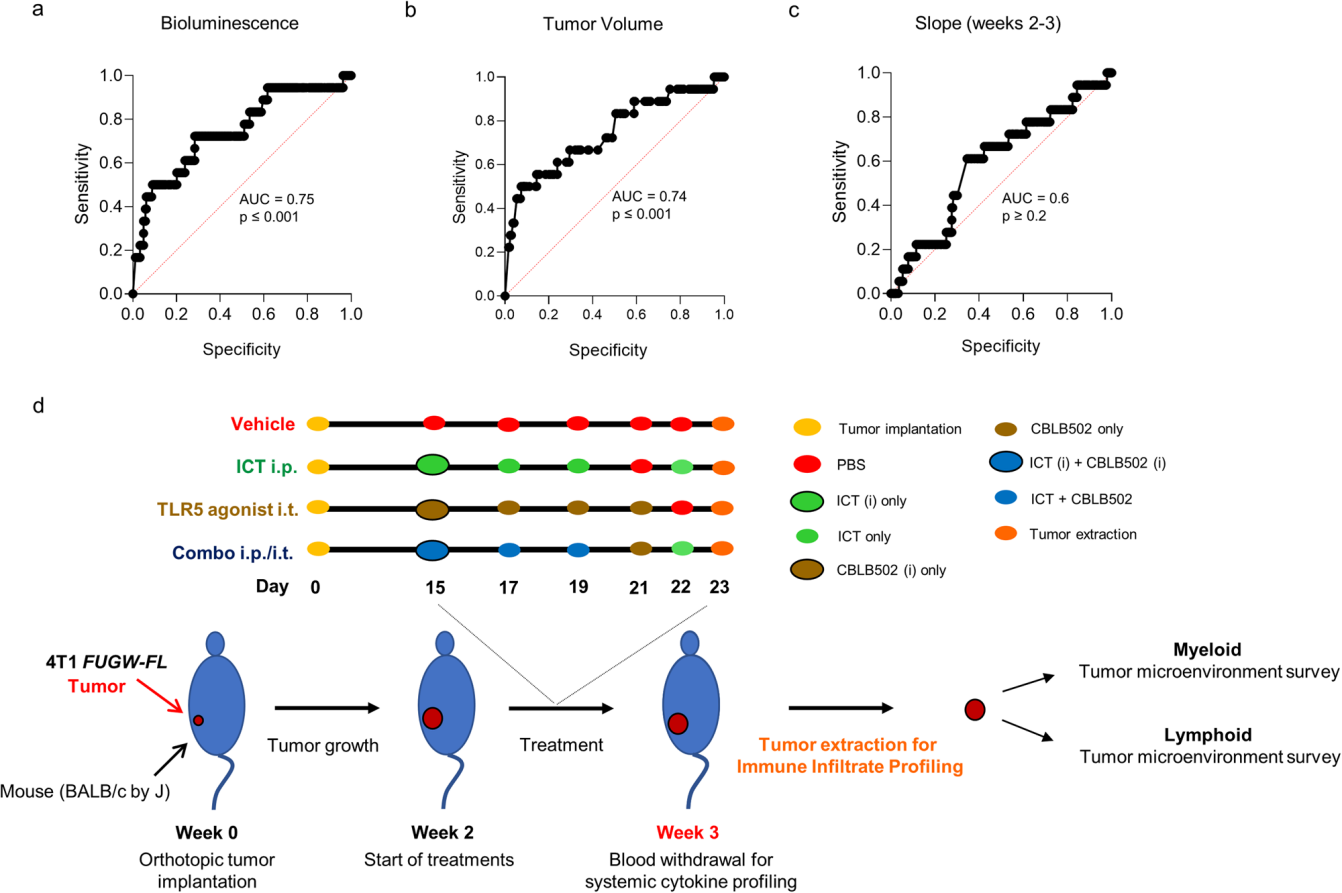
ROC curve and experiment design for cytokine and tumor immune experiment. ROC curves comparing sensitivity and specificity for overall survival by **a** bioluminescence (total photon flux) at week 3, **b** tumor volume at week 3, and **c** tumor-volume slope between weeks 2 and 3. **d** Treatment diagram for euthanized mice used for tumor immune profiling.

Tumor volume and BLI measurements have been useful tools to appraise tumor progression in pre-clinical models^84–87^. Furthermore, BLI measurements have been used to evaluate treatment efficacy in various animal models^88,89^. Hence, we leveraged overall survival data and tumor progression measurements (Fig. 2a, b, and Supplementary Fig. 2) to evaluate the predictive potential for survival outcome of bioluminescence (total photon flux) and/or tumor volume measurements 3 weeks post 4T1 *FUGW-FL* tumor implantation. Area under the curve (AUC) of the receiver operating characteristic (ROC) curve was used to determine the overall diagnostic accuracy of each measurement, dichotomizing the dataset into “survivors” and “non-survivors” based upon their survival to 12 weeks^90^. Figure 5a shows that BLI (Log_10_ total photon flux at week 3) had the best predictive value (AUC = 0.75; 95% CI 0.6 to 0.9; *p* ≤ 0.001) closely followed by tumor size (AUC = 0.74, 95% CI 0.6 to 0.9; *p* ≤ 0.001) (Fig. 5b). Slopes of tumor volume from week 2–3 were also utilized as quantifiers, but they did not reach significance (AUC = 0.6, 95% CI 0.5 to 0.7, *p* ≥ 2) (Fig. 5c). There was also little correlation between Log BLI and tumor burden, suggesting additional information could be provided by each measurement. The predictive diagnostic accuracy of BLI measurements for survival outcomes indicated that mice with higher BLI measurements had decreased likelihood of survival and that mice with lower BLI measurements had increased likelihood of survival.

### Myeloid and lymphoid tumor immune infiltrates

We then proceeded to examine changes to the tumor immune microenvironment in response to treatments at three weeks. 4T1 *FUGW-FL* tumor bearing mice were treated with CBLB502 or ICT alone or in combination for one week and euthanized three weeks post tumor implantation for tumor harvesting as shown in Fig. 5d, scheme, and Supplementary Table 9. Tumors were enzymatically digested and prepared for flow cytometry analysis by staining with antibodies targeting either myeloid cells: dendritic cells, monocytes (Ly6C^+^), monocytes (Ly6C^+^, Ly6G^+^), macrophages, M1-like macrophages, and M2-like macrophages or lymphoid cells: B cells, NK cells, CD3^+^ T cells, CD8^+^ T cells, CD4^+^ T cells, and T regs cells (Supplementary Tables 10, 11). Myeloid and lymphoid cells were identified as described in Supplementary Table 12.

Consistent with prior observations of 4T1 tumors^91,92^ myeloid cells (CD11b^+^ cells) constituted the majority of immune cells (CD45^+^ cells) present in tumor samples taken from vehicle control mice (Supplementary Fig. 9; myeloid cells panel, vehicle control). However, tumor samples taken from treated mice showed a trend toward increased variability in the number of myeloid cells (Supplementary Fig. 9; myeloid cells panel, treated controls). This trend could reflect ongoing immune responses in treated mice. Indeed, overall changes to the tumor immune landscape in treated mice are heterogeneous and may point to immune activation.

**Fig. 6:**
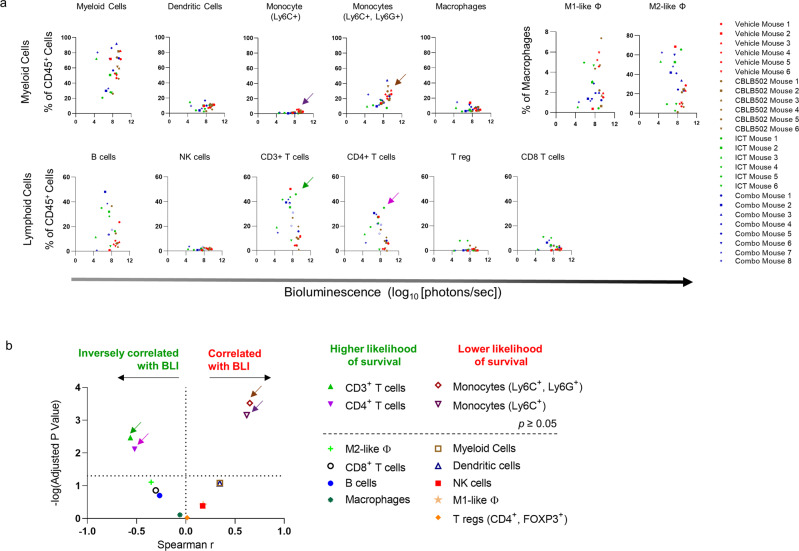
Tumor immune infiltrate profile of vehicle and treated mice, three weeks post orthotopic 4T1 *FUGW-FL* tumor implantation. **a** Myeloid (top panel) and lymphoid (bottom panel) tumor immune infiltrate profile of vehicle and treated mice, assigned a Log BLI measurement taken 3 weeks post 4T1 *FUGW-FL* tumor implantation and prior to tumor extraction. **b** Volcano plot highlighting the correlation between immune infiltrate and Log BLI. Vertical line demarks negative and positive Spearman *r* correlation values. Line of identity indicates *p* = 0.05. Green, light purple, brown, and dark purple arrows highlight CD3^+^ T cells, CD4^+^ T cells, monocytes (Ly6C^+^, Ly6G^+^), and monocytes (Ly6C^+^), respectively, in panels A and B.

First, we observed that an increase in the proportion of monocytes (Ly6C^+^) and monocytes (Ly6C^+^, Ly6G^+^) correlated with increased BLI signal (*r* = 0.6, *p* < 0.001 and r = 0.7, *p* < 0.001) (Fig. 6a, b, dark purple and brown arrows). Moreover, an increase in the proportion of myeloid cells, dendritic cells, M1-like macrophages, T reg, and NK cells also correlated with increased BLI signal, albeit not reaching statistical significance (*r* = 0.4, *p* > 0.08; *r* = 0.3, *p* > 0.08; *r* = 0.2, *p* > 0.4; *r* = 0.01, *p* > 0.9; *r* = 0.2, *p* > 0.4, respectively) (Fig. 6a, b). The increase in the number Ly6C^+^ and Ly6C^+^, Ly6G^+^ monocytes among tumor-bearing mice with increased BLI signal is noteworthy. 4T1 tumors are known to be potent inducers of monocytic MDSCs^93,94^, a heterogenous population of poorly differentiated myeloid cells. MDSCs are known to suppress T cell activation and proliferation, impair natural killer (NK) cells functions, induce immune suppressive T reg cells, and promote pro-tumor inflammatory states by regulating cross talk between tumor cells, mast cells and macrophages^95^. Consistent with these results, we found increased proportion of CD3^+^ T cells and CD4^+^ T cells inversely correlated with increased BLI signal (*r* = −0.6; *p* ≤ 0.01 and *r* = −0.5; *p* ≤ 0.01) (Fig. 6a, b, green and light purple arrows). Furthermore, increases in CD8^+^ T cells, B cells, macrophages, and M2-like macrophages showed an inverse correlation with increased BLI signal, although the correlations did not reach statistical significance (*r* = −0.3; *p* > 0.3, *r* = −0.3; *p* > 0.2, *r* = −0.06; *p* > 0.9; *r* = −0.4; *p* > 0.8, respectively) (Fig. 6a, b). Overall, these results point to a switch in the state of the tumor immune microenvironment from immune suppression to immune activation in mice with lower BLI signals, which elicits a subsequent curative anti-tumor response.

### Characterization of peripheral blood cytokine profile of tumor immune-profiled mice

Finally, we further pursued to corroborate the diagnostic potential of Log (BLI) signal by comparing the blood-borne cytokine profile of long-term survivor mice (Fig. 4a, b) with immune profiled mice (Supplementary Fig. 8a–e and Supplementary Tables 13–16). Consistent with the peripheral blood cytokine profile of long-term survivor mice, euthanized mice with lower Log (BLI) signal correlated with decreased G-CSF (*r* = −0.8; *p* ≤ 0.001) (Supplementary Fig. 10a, b). Of note, CXCL5, previously identified as detrimental for survival (Fig. 4b), did not show statistically significant differences: (*r* = −0.2; *p* > 0.5) (Supplementary Fig. 10a, b). However, it is possible that sample size limited the ability to detect statistical differences.

## Discussion

4T1 mammary carcinoma is a robust murine model to study human triple negative breast cancer, which is highly invasive, metastatic, and resistant to immune check point therapies^96,97^. Herein we report: (1) the successful treatment of established ICT-refractory murine 4T1 mammary carcinoma and the poorly immunogenic and ICT-refractory B16-F10 melanoma tumor model through the combination of standard ICT plus potent innate immune activating TLR5 agonists, (2) host TLR5 receptors are necessary for enhanced survival in mice treated with combination of ICT plus TLR5 agonist in 4T1 tumor-bearing mice, (3) immune-related treatments elicited immune memory against tumor antigens in most long-term survivors, (4) systemic cytokine profiles implicated engagement of both innate and adaptive immunity in response to treatment, (5) CXCL5 and G-CSF may function as a bio-markers for positive responses to treatment, (6) IL-15 points to engagement of adaptive immunity in eliciting a curative anti-tumor response, (7) a novel approach that leveraged non-invasive BLI signal as a mid-point biomarker of tumor progression at week 3 served as a predictive tool for survival outcomes at week 7 or later, which was then used to identify changes in the mid-point tumor immune microenvironment in individual mice that best correlated with increased probability of survival, and (8) decreases in monocytes (Ly6C^+^) and monocytes (Ly6C^+^, Ly6G^+^) accompanied by an increase in CD3 + T cell and CD4 + T cells likely drove the tumor microenvironment state from immune suppression to immune activation. Indeed, because bacterial flagellin is the native ligand for TLR5, overall, our studies provide further mechanistic insight into linkages between ICT response and microbiota^98–101^.

**Fig. 7:**
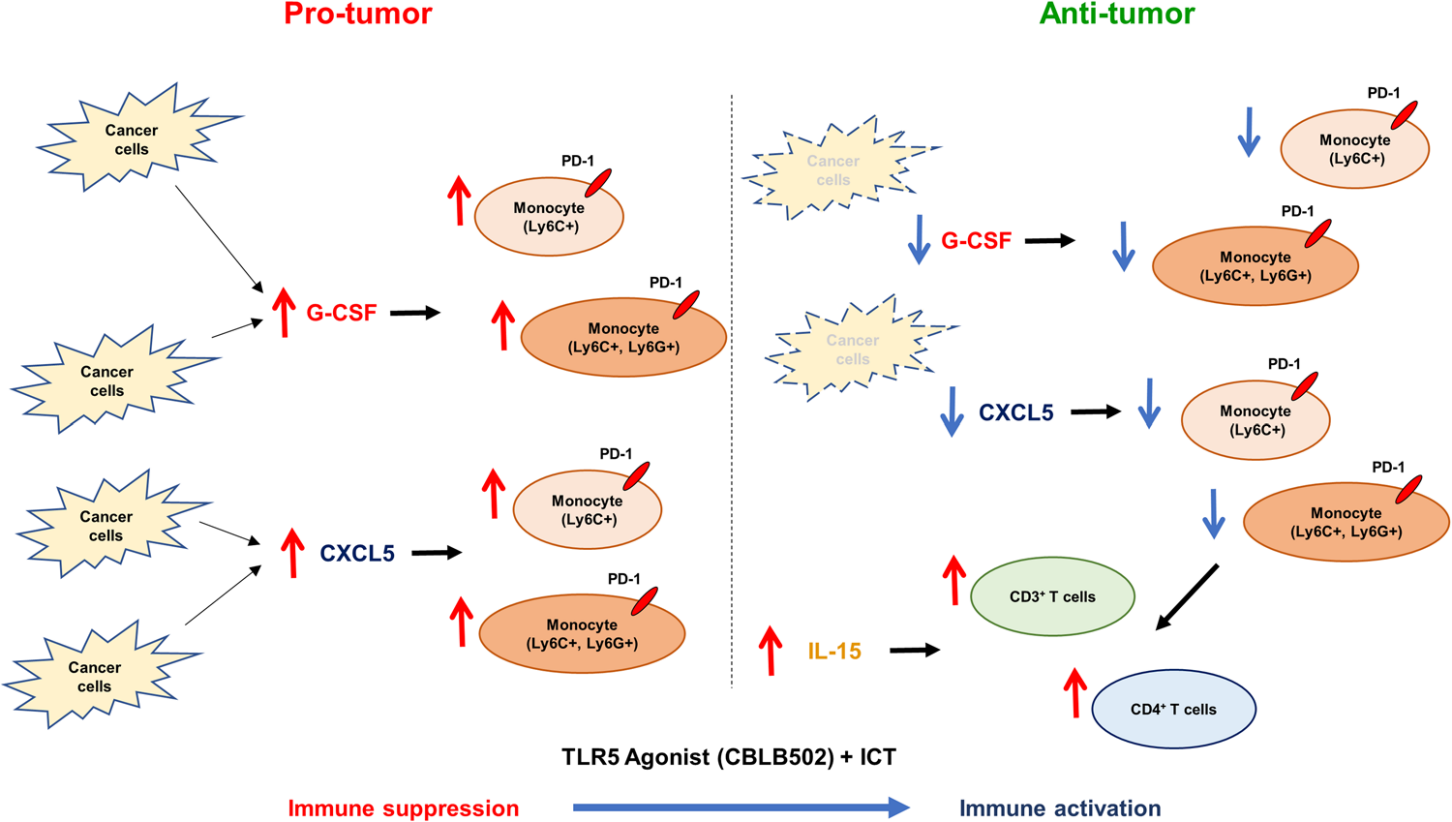
4T1 tumor immune state switches from immune suppression to immune activation in response to combination treatment with TLR5 agonists and ICT. Tumor microenvironment of BALB/c mice implanted with *4T1 FUGW-FL* are highly infiltrated by immune suppressor cells favoring pro-tumor microenvironment (left panel). Potent activation of innate immunity through TLR5 agonists in combination with ICT treatments targeting PD-1 and CTLA-4 leads to systemic increases in immune activating cytokine IL-15, and reduction in immune suppressive cytokines G-CSF and CXCL5. Concomitantly, decreases in monocytes (Ly6C^+^) and monocytes (Ly6C^+^, Ly6G^+^), accompanied by an increase in CD3^+^ T cells and CD4^+^ T cells leads to immune activation and an anti-tumor response (right panel).

Taken together, these results pointed to a new therapeutic strategy that harnessed both innate and adaptive components of the immune system to elicit a lasting antitumor response. On the one hand, bacterial derived-flagellin has been shown to elicit a targeted antitumor response by binding to TLR5, initiating a cascade of signals that produce a pro-inflammatory response via activation of the transcription factor NF-kB^32^. Herein, we first showed that in vitro CBLB502, a potent activator of the NF-kB signaling pathways, was sufficient to elicit a TLR5-mediated immunogenic cytokine response in tumor cells. Given that deficiencies in antigen presentation underlie many mechanisms of resistance against ICT, it is possible to hypothesize that a potent activator of innate immunity may modulate tumor homeostasis, shifting the tumor microenvironment state from immune suppression to immune activation (Fig. 7). Several lines of evidence support this model. First, only treatment with either flagella or CBLB502 in combination with ICT increased survival in mice bearing highly ICT-refractory 4T1 and B16-F10 tumors, whereas monotherapies of flagellin, CBLB502, or ICT did not show significant curative effects. Second, the peripheral blood cytokine profiles of 4T1 tumor-bearing mice that responded to treatment and showed complete tumor regression reflected a concerted antitumor response. Third, *Tlr5*^−/−^ mice failed to respond to combination therapy comprising ICT plus TLR5 agonist, indicating that host TLR5 receptors are necessary for enhanced survival. While it has been shown that mice bearing tumor cells lacking TLR5 fail to respond to treatment with flagellin^26^, it remains to be studied whether flagella and CBLB502 can also act on the tumor cells to elicit a curative immune response in the context of combination treatment with ICT. Furthermore, it remains to be elucidated whether host TLR5 receptors mostly act on components of innate immunity axis or could they also *directly* act on components of adaptive immunity to elicit durable immune responses in the context of ICT^34^. Fourth, nearly all survivor mice that were re-challenged rejected the same tumor, implying an adaptive memory response against tumor cells. Fifth, the peripheral cytokine profiles of those mice that were re-challenged and rejected the tumor aligned with a strong adaptive immune-activating response. Finally, changes in the tumor immune infiltrate amongst mice with increased likelihood of survival were consistent with immune activity leading to a curative anti-tumor response.

The remarkable increase of blood-borne G-CSF protein levels in tumor-bearing mice that either failed treatment or served as tumor-bearing untreated controls suggested that G-CSF may be explored as a potential biomarker. Conversely, low serum level of G-CSF in tumor-bearing mice that responded to treatment or developed long-term tumor immunity suggested utility as a predictive marker for treatment response. These results further raise caution for the use of G-CSF to prevent neutropenia in cancer patients. Although a recent meta-analysis study showed some benefit of supportive G-CSF therapy in overall survival of patients receiving chemotherapy, data also show an increased risk of developing secondary malignancies^102^. G-CSF therapy in the context of ICT remains to be explored. Likewise, it is noteworthy that long-term survivor mice showed statistically significant decreases in CXCL5 compared with mice that failed treatment (non-survivors), indicating that CXCL5 may also be explored as a biomarker for treatment response and/or as a potential therapeutic target.

In conclusion, the success of immune checkpoint therapy in eliciting long lasting curative responses against various types of cancers in subsets of patients make worthwhile efforts to expand the number of patients that respond to this type of treatment. Activators of TLR5, such as flagellin and CBLB502, in combination with ICT may provide new therapeutic opportunities for previously unresponsive patients.

## Methods

### Reagents

*Salmonella typhimurium* flagellin (FLA-ST) was purchased from Invivogen. CBLB502 was a gift from Cleveland Biolabs, Inc. Monoclonal antibodies 9D9 (anti-CTLA-4) and RPM1-14 (anti-PD-1) were purchased from BioX Cell and maintained in 6.5 and 6.7 mg/mL stocks, respectively, and stored at 4 °C before use. *d*-luciferin (*d*-Luc) (BioGold), the substrate for firefly luciferase, was maintained in a 30 mg/mL solution of phosphate-buffered saline (PBS). Matrigel was obtain from Corning and maintained at −20 °C.

### Creation of 4T1 κB_5_:IκBα-FLuc-expressing cells for analysis in vitro

4T1 mammary carcinoma cells (ATCC) at 95% confluency were co-transfected with 10 µg of *pκB*_*5*_*:IκBα-FLuc*^52^ and 3 µg of *pIRES-puro* plasmid DNA using Fugene 6 (Roche) in 10 cm dishes (BD Bioscience). Cultures were incubated at 37 °C and after 24 h, the media was replaced with fresh RPMI supplemented with 10% heat-inactivated FBS. 24 h later, cells were split at multiple dilutions into media containing 0.5 µg/ml puromycin to select for stable transformants. After two weeks, isolated cell colonies were imaged to confirm reporter gene expression, and bioluminescent colonies were harvested and expanded. Reporter cells were continuously cultured in the presence of 0.5 µg/ml puromycin to maintain the expression of the reporter plasmid.

### 4T1 FUGW-FL and B16-F10 cells for analysis in vivo

4T1 mammary carcinoma reporter cells FUGW^103^ stably transfected with the *EF1α:FLuc* plasmid producing constitutive florescent and bioluminescent dual imaging reporter cells (*FUGW-FL*) were cultured according to ATCC protocols and kept under selection with 0.5 µg/ml puromycin^104^. B16-F10 parental cells were cultured according to ATCC protocols^105^.

### Analysis of NF-κB signaling in vitro

4T1 *κB*_*5*_*:IκBα-FLuc* reporter cells (7000 cells) were added to a 96-well plate and incubated overnight at 37 °C. One hour prior to imaging, cell media were aspirated and replaced with RPMI with L-Glutamate (4T1 cells) supplemented with 10% heat-inactivated FBS and 150 µg/ml *d*-luciferin (BioGold). Cells were imaged in an IVIS 100 imaging system, with images being acquired every 5 min for 4 h, unless otherwise indicated. Cells were maintained in the imaging chamber by a heated stage (37 °C) and 5% CO_2_ air flow. Acquisition parameters were: acquisition time, 60 sec; binning, 4–8; filter, open; f stop, 1; FOV, 12–23 cm. Stimuli included: TNFα (20 ng/ml) (R & D systems); flagellin (various concentrations ranging from 1 μg/mL to 0.1 ng/mL); CBLB502 (various concentrations ranging from 1 μg/mL to 0.1 ng/mL); and nuclease-free water (vector only control) added to triplicate wells. Bioluminescence photon flux data (photons/sec) represent the mean of triplicate wells for the indicated number of independent experiments, and were analyzed by region of interest (ROI) measurements with Living Image 3.2 (Caliper Life Sciences). Data were imported into Excel (Microsoft Corp.), averaged, and normalized to both initial (*t* = 0) values (fold-initial) and vehicle-treated controls (fold-vehicle) for presentation in dynamic plots^50^. The normalized results from repeated experiments were averaged for each time point, and the results graphed as normalized photon flux versus time, with the *y*-axis on a log2 scale. Positive error bars represent standard error of the mean for repeated experiments.

### Mice

All animal procedures were approved by the Institutional Animal Care and Use Committee (IACUC) of the University of Texas M.D. Anderson Cancer Center; protocol 00001179-RN01. Female BALB/c mice (4 weeks old) were purchased from The Jackson Laboratory. BALB/c *Tlr5*^−/−^ mating pair of mice were a gift of Joon Haeng Rhee, Chonnam National University Medical School, South Korea^106^. BALB/c *Tlr5*^−/−^ were bred and maintained by the Department of Veterinary Medicine and Surgery of the University of Texas M.D. Anderson Cancer Center. Female C57BL/6J (6–9 weeks old) were purchased from The Jackson Laboratory. Animals were allowed at least one week to acclimate to the animal facility before start of experiments.

### Mouse endpoint protocol

Mice that reached end point (moribund condition or having one tumor measurement in the sagittal or axial plane greater than 1.5 cm) were euthanized, according to University of Texas M.D. Anderson Cancer Center IACUC euthanasia protocols.

### Allograft model of breast cancer

A mammary cell carcinoma allograft was established in BALB/c mice (5–6 week old mice) by orthotopic injection into the fourth mammary fat pad of approximately 10,000 4T1 *FUGW-FL* cells mixed with Matrigel at a 2:1 ratio. The total volume injected into each mouse was 30 μL.

### Administration of flagellin, CBLB502, and immune checkpoint therapy

Flagellin, CBLB502, 9D9 (anti-CTLA-4), and RPM1-14 (anti-PD-1) were suspended in filtered PBS; filtered PBS was used as a vehicle control. Two weeks post orthotopic injection of 4T1 *FUGW-FL* cells into the mammary fat pad, each mouse was randomly sorted into groups receiving vehicle control (*n* = 45, total), or treatment with flagellin only (*n* = 22), CBLB502 only (*n* = 30), ICT only (9D9 plus RPM1-14) (*n* = 37), flagellin combined with ICT or CBLB502 combined with ICT (*n* = 52). Flagellin or CBLB502 was administered every two days for two weeks. All mice sorted into different treatment groups showed detectable levels of bioluminescence signal during week 1 and week 2 post 4T1 *FUGW-FL* tumor cell implantation, confirming the presence of tumors prior to commencement of treatment (Supplementary Figs. 2, 3b–e, 4b–e, 6, and 8b–e). On the first day of treatment, 10 μg of flagellin solution in 50 μL (PBS), or 10 μg (high dose) or 1 μg (low dose) of CBLB502 solution in 50 μL (PBS) were administered as an intra-tumoral or intraperitoneal injection into designated animals. For each subsequent treatment, 2 μg of flagellin solution in 50 μL (PBS) or 2 μg (high dose) or 200 ng (low dose) of CBLB502 solution in 50 μL (PBS) was used. ICT was administered on days 1, 3, 5, and 8 of treatment. On the first day, mice receiving ICT treatment were injected intraperitoneally with 200 μg in 100 μL of both 9D9 and RPM1-14 (200 μL total per mouse). On subsequent days, each mouse was injected with 100 μg in 100 μL of each antibody. On days when both flagellin and ICT were administered, vehicle control mice were given two intraperitoneal injections of 100 μL PBS or one intraperitoneal injection of 200 μL PBS and one intra-tumoral injection of 50 μL PBS. On days when only flagellin was administered, vehicle mice received only 50 μL PBS intra-tumoral injection, and on the day that only ICT was administered, they received only two 100 μL intraperitoneal injections of PBS or one intraperitoneal injection of 200 μL PBS.

### Bioluminescence imaging in vivo

The mice were imaged using the PerkinElmer IVIS Spectrum Imaging System weekly beginning one week after orthotopic injection of 4T1 *FUGW-FL* cells into the mammary fat pad. The mice were weighed at the beginning of each imaging session, and 165 μg *d*-luciferin (prepared at 30 mg/mL in PBS) was injected intraperitoneally per gram of mouse. Mice were imaged ten minutes after injection with *d*-luciferin^50^.

### Confirmation of Tlr5^+/+^ and Tlr5^−/−^ mice genotypes

Genomic DNA from *Tlr5*^+/+^ and *Tlr5*^−/−^ mice was obtained by clipping less than 3 mm tail tip from mice. Tail samples were processed using DirectPCR Lysis Reagent Tail (Viagen Biotech Inc), followed by PCR (Supplementary Fig. 11a, b). The genomic region containing the *Tlr5* gene was amplified using the following primers and PCR protocol:

***Tlr5*-WT:** 5′-CTA TCT GGC AAC CAG ATT CAC AGC CTC-3′

***Tlr5*-KO:** 5′-CTA AAG CGC ATG CTC CAG ACT GCC TTG-3′

***Tlr5*-extra:** 5′-CAG GTC GTT AAA TAT CCC AGG TGG AAG-3′

### PCR protocol

Initial denaturation, 94 °C for 5 min; denaturation, 94 °C for 1 min; annealing, 62 °C for 1 min; extension, 72 °C for 1 minute, 30 cycles followed by a final extension, 72 °C for 5 min.

### Allograft model of melanoma

A melanoma tumor was established in C57BL/6J mice (Jackson Laboratory, 6–9 week old mice) by subcutaneous injection into the right dorsal flank of approximately 12,000 B16-F10 cells. The total volume injected into each mouse was 50 μL of cells resuspend in RPMI 1640 with L-glutamine media (Millipore Sigma).

### Administration of CBLB502 and ICT

Three days post subcutaneous injection of B16-F10 cells into the posterior right flank, each mouse was randomly sorted into a group receiving treatment with vehicle control, CBLB502 only, ICT only (9D9 plus RPM1-14), or CBLB502 combined with ICT. CBLB502, 9D9, and RPM1-14 were suspended in filtered PBS, and filtered PBS was used as a vehicle control. CBLB502 was administered every two days for two weeks. On the first day of treatment, 1 μg in 50 μL of CBLB502 was administered as an intra-tumoral injection into designated animals. For each subsequent treatment, 200 ng in 50 μL of CBLB502 was used. ICT was administered on days 1, 3, 5, and 8 of treatment. On the first day, mice receiving ICT treatment were injected intraperitoneally with 200 μg in 100 μL of both 9D9 and RPM1-14 (200 μL total per mouse). On subsequent treatment days, each mouse was injected with 100 μg in 100 μL of each antibody. On days when both CBLB502 and ICT were administered, vehicle control mice were given an intraperitoneal injection of 200 μL PBS and one intra-tumoral injection of 50 μL PBS. On days when only CBLB502 was administered, vehicle mice received only 50 μL PBS intra-tumoral injection, and on the day that only ICT was administered, they received only a 200 μL intraperitoneal injections of PBS.

### Caliper measurement of tumor volume

Tumor volume was determined by measuring length (*l*, longest measurement) and width (*w*) for each tumor at least once a week by caliper, using the standard triangular prism formula for volume: *V* = (*l* × *w*^2^)/2.

### Cytokine profile in vitro

4T1 *FUGW-FL* florescent and bioluminescent dual reporter cells were plated in 100 mm tissue culture plates (BD) (750,000 cells per plate) and incubated overnight at 37 °C with RPMI supplemented with 10% heat-inactivated FBS. On day two, cell media were aspirated and replaced with RPMI without 10% heat-inactivated FBS. On day three, cultures were treated with either CBLB502 at 1 µg/ml or PBS as vector control in triplicates. On day four, media was collected into 15 ml tubes, centrifuge at 2000 rpm at 4 °C for 10 min. The supernatant was assayed using Mouse Cytokine Antibody Array C series 1000 (RayBiotech).

### Cytokine profile in vivo

Sera from mice in experiments 6, 7, and 8 (Supplementary Table 2) were obtained by submandibular sampling. Samples were allowed to clot at room temperature for 1 hour, centrifuge at 2000 × *g* for 10 min at room temperature and sera (upper phase) was collected into Eppendorf tubes. Sera from mice in experiment 9 (Supplementary Table 2) were obtained by saphenous sampling. Samples were collected into serum separation tubes (Thermo-Fisher), centrifuged at 2000 × *g* for 10 min at 4 °C and sera (upper phase) were collected into Eppendorf tubes. All serum samples were stored at −80 °C. Blood samples did not exceed 10% of total circulating blood volume^107^.

### Luminex multiplex quantitative analysis

Serum samples were analyzed at the Antibody-Based Proteomics Core at Baylor College of Medicine, Houston, TX. The core used the Milliplex Mouse 32-Plex Cytokine Panel (Millipore), which included the following cytokines: G-CSF, GM-CSF, CCL11, IFN-γ, IL-1α, IL-1β, IL-2, IL-3, IL-4, IL-5, IL-6, IL-7, IL-9, IL-10, IL-12 (p40), IL-12 (p70), IL-13, IL-15, IL-17, CXCL10, CXCL1-like, LIF, CXCL5, CCL2, M-CSF, CXCL9, CCL3, CCL4, CXCL2, CCL5, TNF-α, VEGF and appropriate controls and calibration standards. For cytokines with above-range values, we reported the highest observed value for the corresponding cytokine, whereas for below-range values, we reported the lowest value on the standard curve divided by half^108,109^.

### Tumor immune infiltrate profile

Tumors and spleens were removed with scissors or forceps and weighted. Tumors were chopped into fine pieces and transferred into RPMI media (5% FBS + 10 mM HEPES) containing collagenase (0.2 mg/mL) (*Clostridium histolyticum*, Sigma) and spleens were suspended in PBS. Tumors were shaken gently at 37 °C for 30 min prior to tissue disruption. Spleen and tumor tissues were disrupted using a cell strainer (70 µm nylon) (Corning) using a syringe plunge. Nylon mesh was rinsed several times with media. Samples were spin (5 min at 1500 rpm) and re-suspended in 1 mL RBC lysis buffer (Sigma) for 5 min and washed 2× with flow buffer (Thermo Fisher). Cells were counted (Nexelom Cellometer), prior to live/dead staining with Zombie UV (Biolegend), incubated for 15 min at room temperature and washed 2× with PBS. This was followed by Fc block (CD16/CD32, BD Biosciences), antibody staining and preparation of single-color compensation controls (Ultra Comp eBeads compensation beads, Thermo Fisher). Samples were covered in foil and kept at 4 °C overnight. Intracellular staining was performed using Permeabilization Buffer (eBioscience) in conjunction with Foxp3/Transcription Factor Staining Buffer set (eBioscience). Fluorescence minus one (FMO) controls were used where indicated to distinguish between positively and negatively stained cells for FoxP3, Ly6G, and Ly6C.

### Antibody cocktail for myeloid panel A

CD45 Brilliant Violet 650 (30-F11, Biolegend), CD11b Pe-Cy7 (M1/70, Biolegend), CD11c Brilliant Violet 711 (N418, Biolegend), F4/80 PE-dazzle 594 (BM8, Biolegend), Ly-6G APC-Fire 750 (1A8, Biolegend), Ly-6C Brilliant Violet 510 (HK1.4, Biolegend), CD80 Brilliant Violet 650 (16-10A1, Biolegend), CD163 PerCP-eFluor 710 (TNKUPL, Biolegend), and CD285 PE (TLR-5) (ACT5, BD Biosciences) (Supplementary Table 10).

### Antibody cocktail for lymphoid panel A

CD45 Alexa Fluor 700 (30-F11, Biolegend), CD19 PerCP/Cy5 (6D5, Biolegend), CD3ε Brilliant Violet 650 (17A2, Biolegend), CD4 PerCP-Cy5.5 (GK1.5, Biolegend), CD49b PE-dazzle 594 (DX5, Biolegend), CD8a Brilliant Brilliant Violet 510 (53–6.7, Biolegend), FoxP3 Alexa 647 (3G3, Thermo-Fisher), and CD285 PE (TLR-5) (ACT5, BD Biosciences) (Supplementary Table 10).

### Antibody cocktail for myeloid panel B

CD45 Alexa 700 (30-F11, Biolegend), CD11b PerCP/Cyanine5.5 (M1/70, Biolegend), CD11c Brilliant Violet 510 (N418, Biolegend), F4/80 Brilliant Violet 785 (BM8, Biolegend), Ly-6G PE/Cy7 (1A8, Biolegend), Ly-6C Brilliant Violet 605 (HK1.4, Biolegend), CD80 Brilliant Violet 421 (16-10A1, Biolegend), CD163 APC (S15049I, Biolegend), and CD285 PE (TLR-5) (ACT5, BD Biosciences) (Supplementary Table 11).

### Antibody cocktail for lymphoid panel B

CD45 Alexa 700 (30-F11, Biolegend), CD19 PerCP/Cy5.5 (1D3/CD19, Biolegend), CD3ε Brilliant Violet 510 (17A2, Biolegend), CD4 PE/Cy7 (GK1.5, Biolegend), CD49b BV421 (DX5, Biolegend), CD8a Brilliant Brilliant Violet 711 (53–6.7, Biolegend), FoxP3 Alexa 647 (MF-14, Biolegend), and CD285 PE (TLR-5) (ACT5, BD Biosciences) (Supplementary Table 11).

### Flow cytometry

Flow cytometry was performed using an LSRII cytometer (Becton Dickinson). Subsequent analysis was performed utilizing FlowJo 10.7.1 (FlowJo, Becton Dickinson).

### Statistics and reproducibility

Statistical analyses were performed using GraphPad Prism version 8.0.0 for Windows, GraphPad Software, San Diego, California. Overall survival was assessed using Kaplan–Meier curves^110^. Log-rank (Mantel–Cox) test and Gehan–Breslow–Wilcoxon tests were used to compare survival rates between treatments. ROC curves were calculated for predictive analysis. Two way Anova and multi *t*-test comparisons using the Holm–Sidak method, with alpha = 0.05, were used to determine detectable statistical difference in in vitro and in vivo cytokine studies. Pearson correlation coefficient (*r*) was calculated to assess correlation between likelihood of survival and proportion of tumor immune infiltrate; a two tailed *p* value ≤ 0.05 was used to determine detectable statistical significance.

### Reporting summary

Further information on research design is available in the Nature Portfolio Reporting Summary linked to this article.

## Supplementary information

Supplemental Material

Description of Additional Supplementary Files

Supplementary Data 1

Reporting Summary

## Data Availability

All data generated or analyzed during this study are included in this published article and its supplementary information files, including Supplementary Data 1.
