# Supplementary material for: TLR5 agonists enhance anti-tumor immunity and overcome resistance to immune checkpoint therapy
Source: Commun Biol. 2023 Jan 12;6:31. doi: 10.1038/s42003-022-04403-8 (PMC9837180; doi:10.1038/s42003-022-04403-8)
Supplement: Supplementary file 1 — Supplemental Material [file 42003_2022_4403_MOESM1_ESM.pdf]

Supplemental Material

TLR5 Agonists Enhance Anti-Tumor Immunity and Overcome Resistance to Immune Checkpoint Therapy

Caleb Gonzalez[1], Sarah Williamson[1], Seth T. Gammon[1], Sarah Glazer[1], Joon Haeng Rhee[2], and David Piwnica-Worms[1]✉

[1]Department of Cancer Systems Imaging, University of Texas

MD Anderson Cancer Center, Houston, TX 77030, USA

[2]Chonnam National University Medical School,

Gwangju, South Korea

Keywords: Cancer, immune checkpoint therapy, flagellin, CBLB502, TLR5, NF-κB, luciferase, bioluminescence reporter

✉Corresponding author:
David Piwnica-Worms, M.D., Ph.D.,
Department of Cancer Systems Imaging,
The University of Texas M.D. Anderson Cancer Center,
1400 Pressler Street, Unit 1479
FCT16.6030, Houston, Texas 77030
Tel: 713-745-0850
Fax: 713-745-7540
Email: dpiwnica-worms@mdanderson.org

a

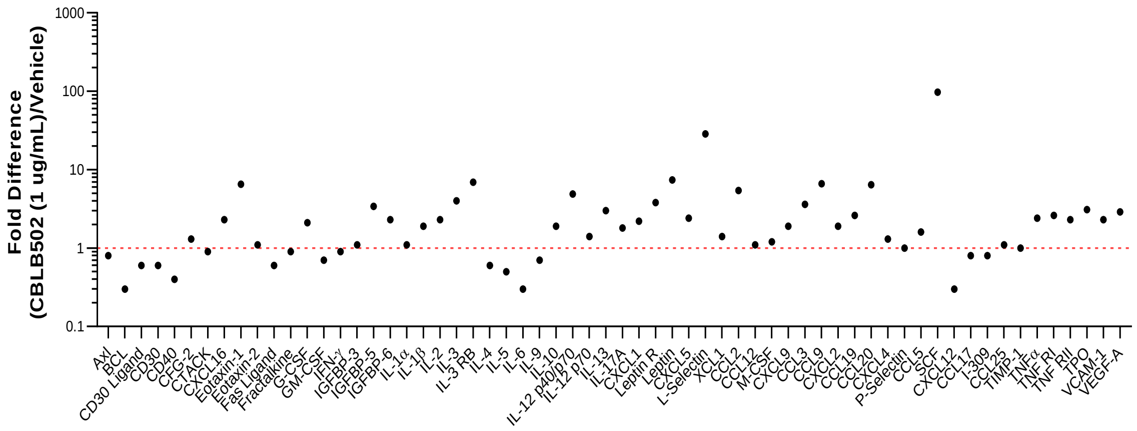

b

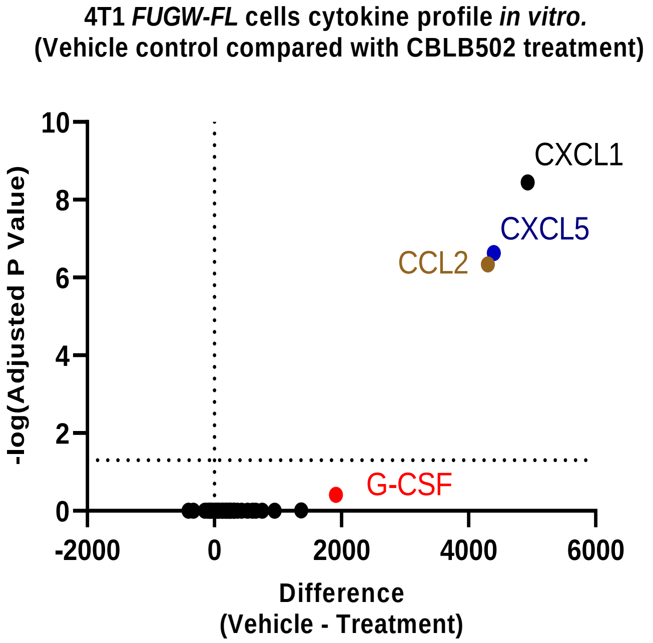

Supplementary Figure 1. (a) Cytokine profile *in vitro* of 62 cytokines secreted from 4T1 *FUGW-FL* cells in response to overnight treatment with vehicle control (PBS) or CBLB502 (1 µg/mL). Data are shown as normalized signal intensity (SI) of the mean value of three membranes per treatment. SI was measured with ImageJ (NIH, Maryland, USA). Normalized SI were calculated using the following equation N = (A-C) * M/T, where N is normalized antibody SI, A is the antibody SI, measured through ImageJ, NIH, C is the average of the negative control spot SI and blank spot SI, M is the positive control SI of a reference membrane (chosen arbitrarily), T is the SI of the positive control of the membrane of the sample. (b) Volcano plot highlighting detectable statistical differences between 4T1 *FUGW-FL* cells treated with CBLB502 (1 µg/mL) and vehicle control (PBS).

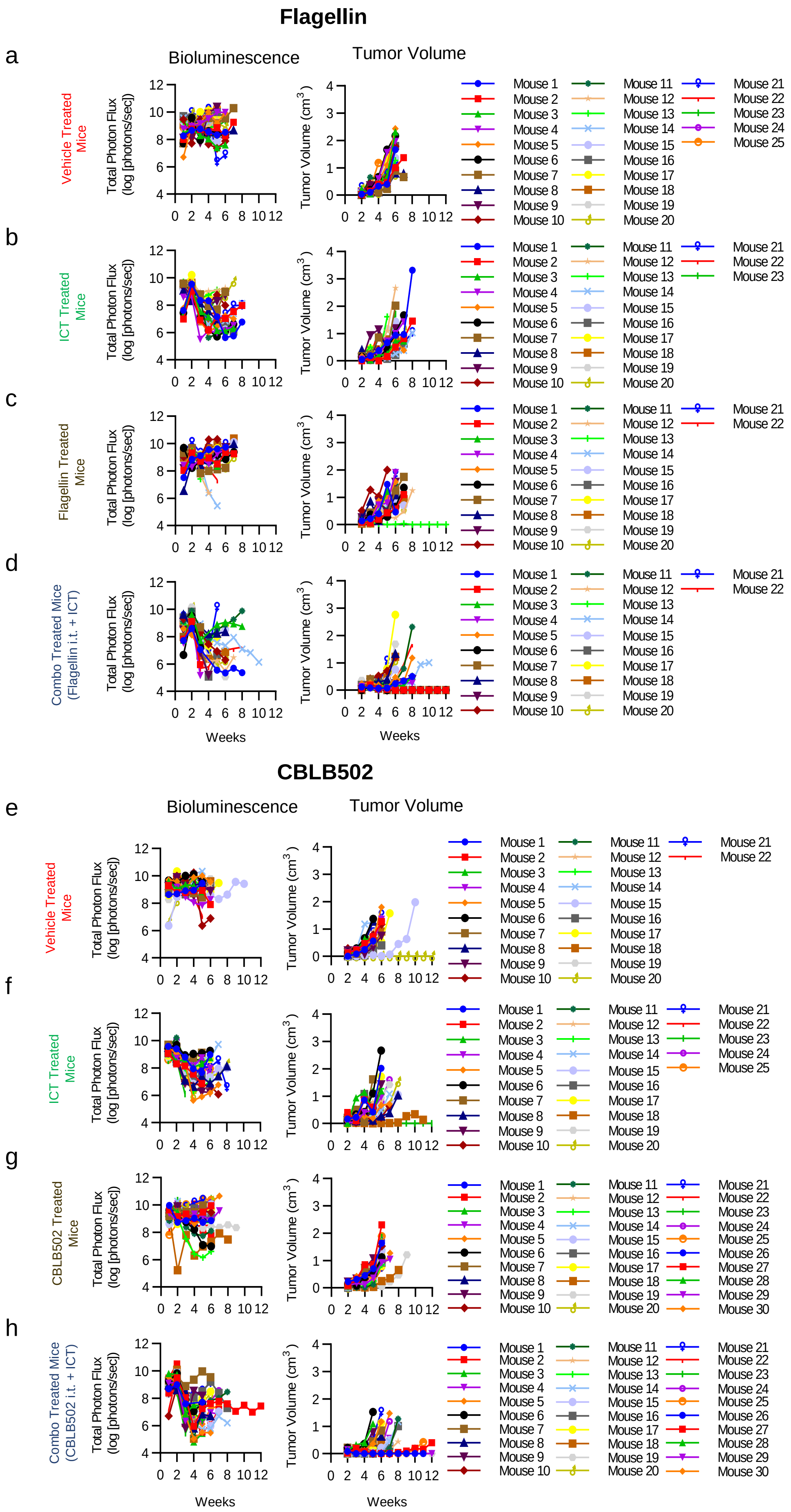

Flagellin
a
Bioluminescence
Tumor Volume
Vehicle Treated Mice
b
ICT Treated Mice
c
Flagellin Treated Mice
d
Combo Treated Mice (Flagellin i.t. + ICT)
Total Photon Flux (log [photons/sec])
Tumor Volume (cm³)
Weeks
CBLB502
e
Bioluminescence
Tumor Volume
Vehicle Treated Mice
f
ICT Treated Mice
g
CBLB502 Treated Mice
h
Combo Treated Mice (CBLB502 i.t. + ICT)
Total Photon Flux (log [photons/sec])
Tumor Volume (cm³)
Weeks
Mouse 1
Mouse 2
Mouse 3
Mouse 4
Mouse 5
Mouse 6
Mouse 7
Mouse 8
Mouse 9
Mouse 10
Mouse 11
Mouse 12
Mouse 13
Mouse 14
Mouse 15
Mouse 16
Mouse 17
Mouse 18
Mouse 19
Mouse 20
Mouse 21
Mouse 22
Mouse 23
Mouse 24
Mouse 25
Mouse 26
Mouse 27
Mouse 28
Mouse 29
Mouse 30

Supplementary Figure 2. Tumor progression measurements. (a) Tumor size of vehicle-treated negative control mice (n=25) measured by bioluminescence imaging (total photon flux, left panel) and caliper measurements (tumor volume, right panel). All vehicles-treated mice died by week 8. (b) Tumor size of ICT-treated mice (n=23) measured by bioluminescence imaging (total photon flux, left panel) and caliper measurements (tumor volume, right panel). All ICT-treated mice died by week 8. (c) Tumor size of flagellin-treated mice (n=22) measured by bioluminescence imaging (total photon flux, left panel) and caliper measurements (tumor volume, right panel). One flagellin-treated mouse, Supplementary Fig. 2c-mouse 13, was tumor-free for 40 weeks before it was re-challenge with 4T1 *FUGW-FL* tumor (Supplementary Table 5). (d) Tumor size of flagellin plus ICT-treated mice (n=22) measured by bioluminescence imaging (total photon flux, left panel) and caliper measurements (tumor volume, right panel). Supplementary Fig. 2d-mouse 2, Supplementary Fig. 2d -mouse 6, Supplementary Fig. 2d -mouse 7 were tumor-free for 54, 51, and 51 weeks, respectively, before they were re-challenge with 4T1 *FUGW-FL* tumor cells (Supplementary Table 5). (e) Tumor size of vehicle-treated negative control mice (n=22) measured by bioluminescence imaging (total photon flux, left panel) and caliper measurements (tumor volume, right panel). One vehicle control mouse, Supplementary Fig. 2e-mouse 20, was tumor-free for 18 weeks before it was re-challenge with 4T1 *FUGW-FL* tumor (Supplementary Table 5). (f) Tumor size of ICT-treated mice (n=25) measured by bioluminescence imaging (total photon flux, left panel) and caliper measurements (tumor volume, right panel). One ICT-treated mouse, Supplementary Fig. 2f-mouse 23, was tumor-free for 22 weeks before it was re-challenge with 4T1 *FUGW-FL* tumor (Supplementary Table 5). (g) Tumor size of CBLB502-treated mice (n=30) measured by bioluminescence imaging (total photon flux, left panel) and caliper measurements (tumor volume, right panel). All CBLB502-treated mice died by week 9. (h) Tumor size of CBLB502 plus ICT-treated mice (n=30) measured by bioluminescence imaging (total photon flux, left panel) and caliper measurements (tumor volume, right panel). Supplementary Fig. 2h-mouse 1, Supplementary Fig. 2h -mouse 4, Supplementary Fig. 2h -mouse 10, Supplementary Fig. 2h -mouse 19, Supplementary Fig. 2h-mouse 22, and Supplementary Fig. 2h -mouse 26 were tumor-free for 40, 40, 38, 22, 18 and 18 weeks, respectively, before they were re-challenge with 4T1 *FUGW-FL* tumor cells (Supplementary Table 5).

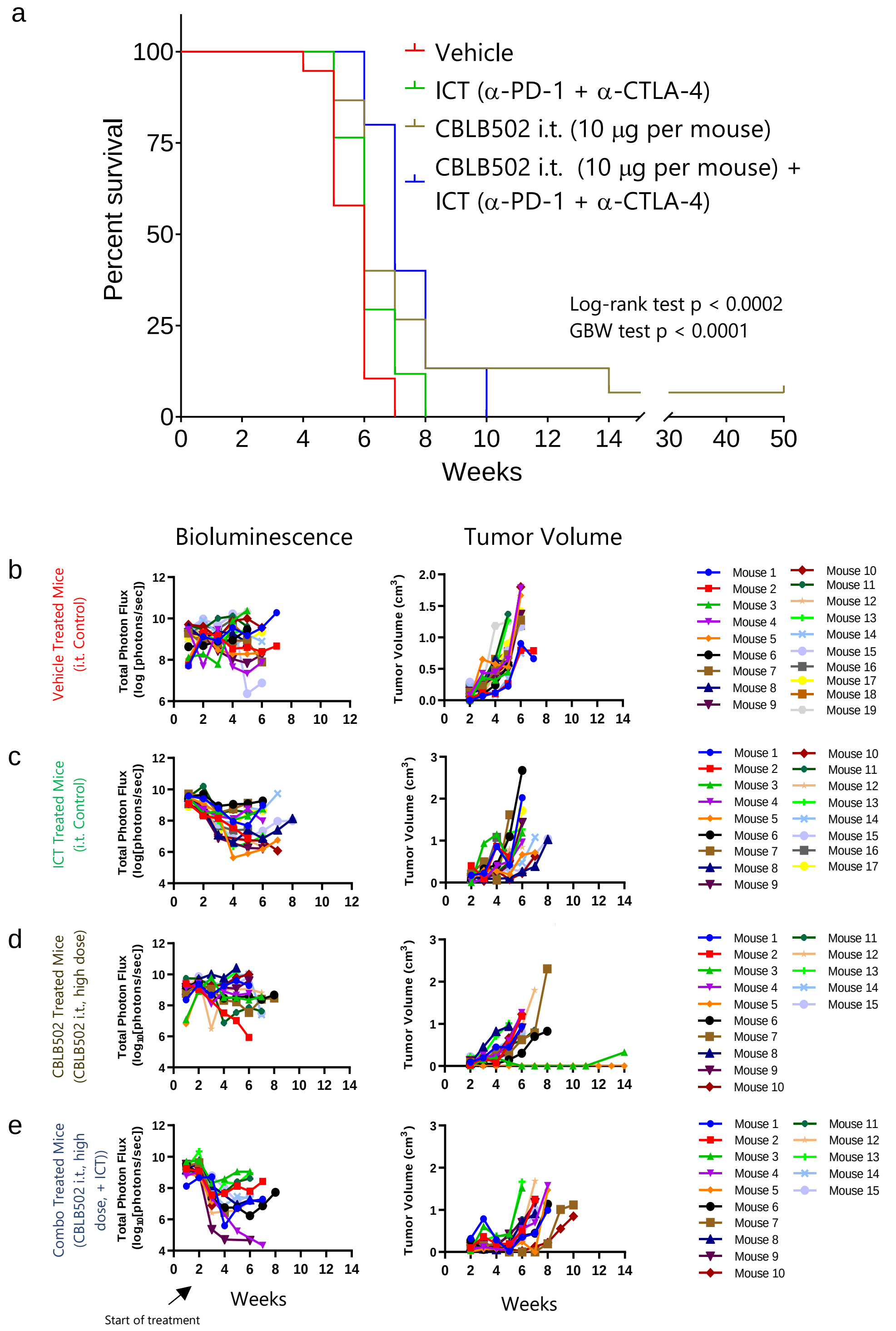

a
Vehicle
ICT (α-PD-1 + α-CTLA-4)
CBLB502 i.t. (10 μg per mouse)
CBLB502 i.t. (10 μg per mouse) + ICT (α-PD-1 + α-CTLA-4)
Percent survival
Weeks
Log-rank test p < 0.0002
GBW test p < 0.0001
Bioluminescence
Tumor Volume
b
Vehicle Treated Mice (i.t. Control)
c
ICT Treated Mice (i.t. Control)
d
CBLB502 Treated Mice (CBLB502 i.t., high dose)
e
Combo Treated Mice (CBLB502 i.t., high dose, + ICT))
Total Photon Flux (log [photons/sec])
Tumor Volume (cm³)
Weeks
Start of treatment

Supplementary Figure 3. Higher CBLB502 dose combined with ICT treatment does not enhance survival in BALB/c mice implanted with orthotopic 4T1 *FUGW-FL* tumor cells. (a) Kaplan-Meier survival analysis of BALB/c mice implanted with orthotopic 4T1 cells stably transfected with EF1a FUGW florescent and bioluminescent reporter (4T1 *FUGW-FL*) at week 0 and treated with CBLB502 high dose with or without ICT from week 2 to week 4. Mice treated with vehicle control (PBS) (n = 19), ICT (n = 17), CBLB502 (n = 15) and CBLB502 in combination with ICT (n=15) were compared. ICT alone did not show detectable difference (p = 0.1, Log-rank test; p = 0.1, Gehan-Breslow-Wilcoxon test). One mouse treated only with CBLB502 (high dose) was tumor-free for 51 weeks post orthotopic tumor implantation (p = 0.01, Log-rank test; p = 0.01 Gehan-Breslow-Wilcoxon test). Treatment with a combination of CBLB502 and ICT did not result in long-term survivors, but had a statistical detectable effect on survival (p = 0.0001, Log-rank test; p = 0.0001, Gehan-Breslow-Wilcoxon test) and when compared with vehicle control the median survival shifted by one week. (b) Tumor size of vehicle-treated negative control mice (n=19) by bioluminescence imaging (total photon flux, left panel) and caliper measurements (tumor volume, right panel). All vehicle control mice were dead by week 7. (c) Tumor size of ICT treated mice (n=17) measured by bioluminescence imaging (total photon flux, left panel) and caliper measurements (tumor volume, right panel). All ICT control mice were dead by week 8. (d) Tumor size of CBLB502 high dose treated mice (n=15) measured by bioluminescence imaging (total photon flux, left panel) and caliper measurements (tumor volume, right panel). One CBLB502 high dose-treated mice was tumor-free for 51 weeks post orthotopic tumor implantation, before it was re-challenge with 4T1 *FUGW-FL* tumor (Supplementary Fig. 3d-mouse 5 and Supplementary Table 5). (e) Tumor size of CBLB502 high dose in ICT treated mice (n=15) by bioluminescence imaging (total photon flux, left panel) and caliper measurements (tumor volume, right panel). All CBLB502 high dose treated with ICT died by week ten. However, two mice showed delayed tumor growth: mouse 7 and 10.

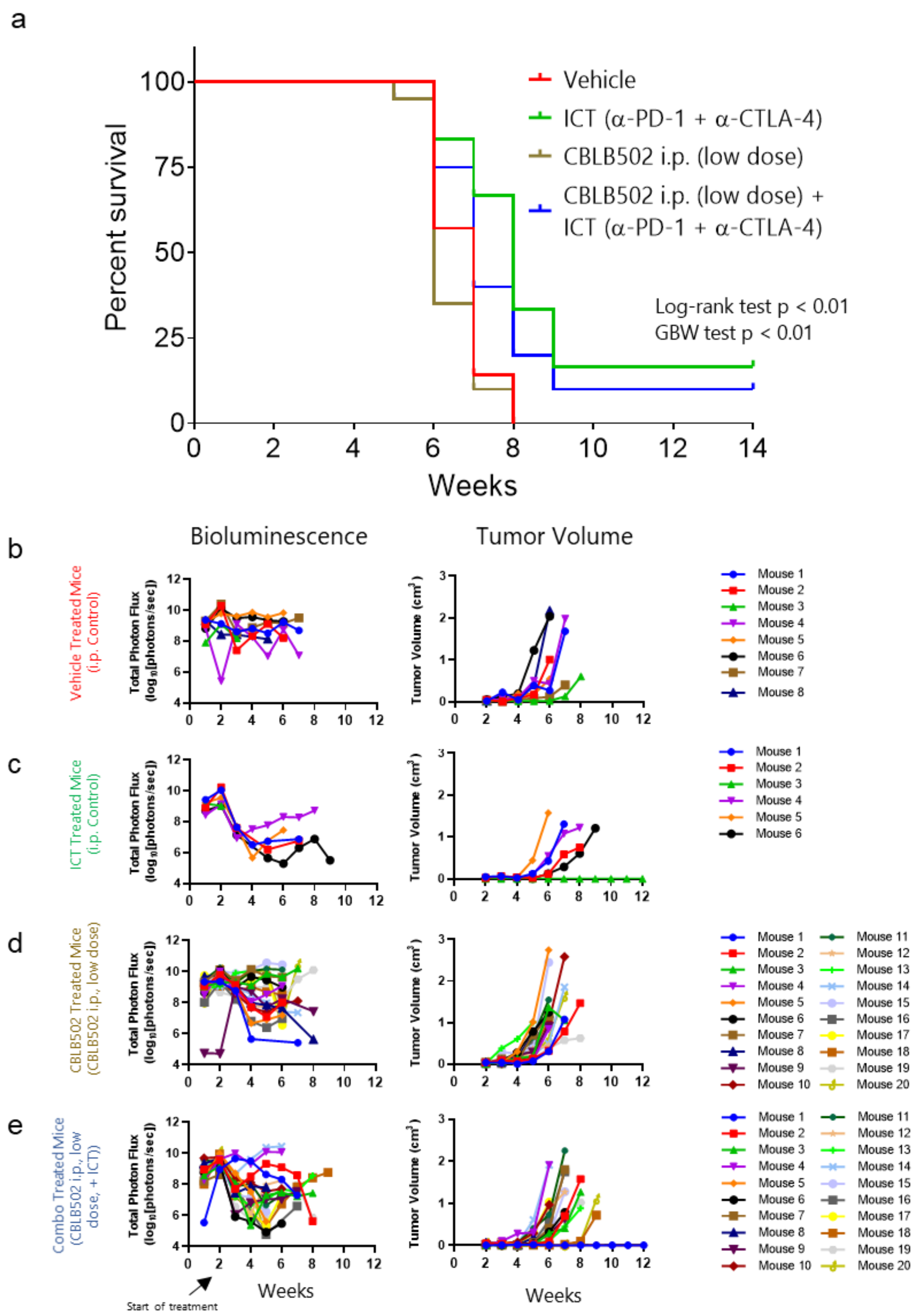

a
Percent survival
Weeks
Vehicle
ICT (α-PD-1 + α-CTLA-4)
CBLB502 i.p. (low dose)
CBLB502 i.p. (low dose) + ICT (α-PD-1 + α-CTLA-4)
Log-rank test p < 0.01
GBW test p < 0.01
b
c
d
e
Bioluminescence
Tumor Volume
Vehicle Treated Mice (i.p. Control)
ICT Treated Mice (i.p. Control)
CBLB502 Treated Mice (CBLB502 i.p., low dose)
Combo Treated Mice (CBLB502 i.p., low dose, + ICT)
Total Photon Flux (log10[photons/sec])
Tumor Volume (cm3)
Weeks
Start of treatment

Supplementary Figure 4. Systemic administration of CBLB502 in BALB/c mice implanted with orthotopic 4T1 *FUGW-FL* tumor cells. (a) Kaplan-Meier survival analysis of BALB/c mice implanted with orthotopic 4T1 *FUGW-FL* cells at week 0 and treated with CBLB502 administered through intraperitoneal injection (i.p.) with or without ICT treatment from week 2 to week 4. Mice treated with vehicle control (PBS) (n = 8), ICT (n = 6), CBLB502 (n = 20) and CBLB502 i.p in combination with ICT (n=20) were compared. Treatment with a combination of CBLB502 i.p. with ICT had a statistically significant effect on survival (p = 0.01, Log-rank test; p = 0.01, Gehan-Breslow-Wilcoxon test). ICT only and CBLB502 i.p. only treatments did not show detectable difference (p = 0.1, Log-rank test; p = 0.1 Gehan-Breslow-Wilcoxon test and p = 0.4 and Log-rank test; p = 0.3 Gehan-Breslow-Wilcoxon test, respectively) when compared to vehicle control. (b) Tumor size of vehicle-treated negative control mice (n=8) by bioluminescence imaging (total photon flux, left panel) and caliper measurements (tumor volume, right panel). All vehicle control mice were dead by week 8. (c) Tumor size of ICT treated mice (n=6) measured by bioluminescence imaging (total photon flux, left panel) and caliper measurements (tumor volume, right panel). One ICT mouse, Supplementary Fig. 4c-mouse 3 was tumor-free for 18 weeks post orthotopic tumor implantation, before it was re-challenge with 4T1 *FUGW-FL* tumor (Supplementary Table 5). (d) Tumor size of CBLB502 i.p. treated mice (n=20) measured by bioluminescence imaging (total photon flux, left panel) and caliper measurements (tumor volume, right panel). All CBLB502 i.p. control mice were dead by week 8. (e) Tumor size of CBLB502 i.p. with ICT treated mice (n=20) measured by bioluminescence imaging (total photon flux, left panel) and caliper measurements (tumor volume, right panel). Two CBLB502 (i.p.) treated with ICT, Supplementary Fig. 4e-mouse 1 and Supplementary Fig. 4e-mouse 5 were tumor-free for 22 weeks post orthotopic tumor implantation, before they were re-challenge with 4T1 *FUGW-FL* tumor (Supplementary Table 5).

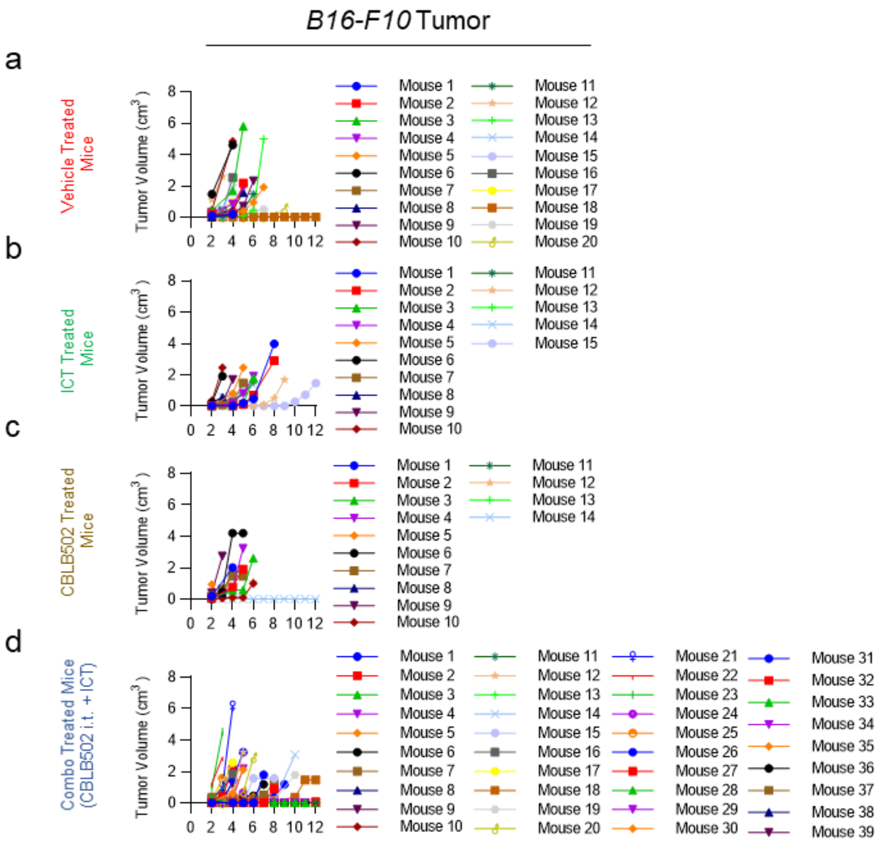

Supplementary Figure 5. Tumor progression measurements of C57BL/6J mice implanted with B16-F10 tumor cells. (a) Tumor size of vehicle-treated negative control mice (n=20) assessed by caliper measurements (tumor volume). One vehicle control mouse, mouse 18, was tumor-free for 12 weeks. (b) Tumor size of ICT-treated mice (n=15) assessed by caliper measurements (tumor volume). All ICT-treated mice died by week 7. (c) Tumor size of CBLB502-treated mice (n=14) assessed by caliper measurements (tumor volume). One CBLB502 treated mouse, mouse 14, was tumor-free for 12 weeks. (d) Tumor size of CBLB502 plus ICT-treated mice (n=39) assessed by caliper measurements (tumor volume). Mice 3, 4, 6, 8, 9, and 10 were tumor-free for at least 77 weeks; mice 11 and 13 were tumor-free for at least 58 weeks; mice 33, 34, 35 and 38 were tumor-free for at least 12 weeks.

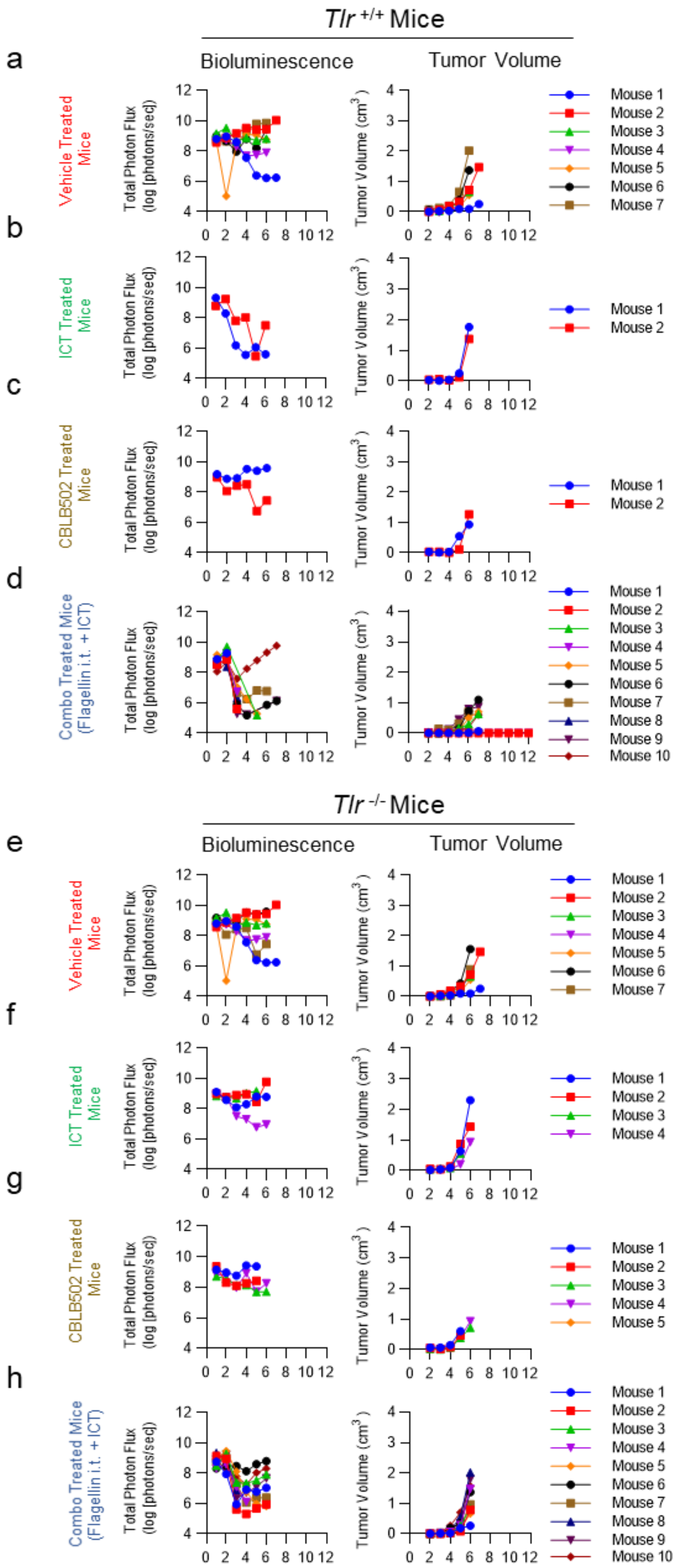

Tlr +/+ Mice
Bioluminescence
Tumor Volume
a
Vehicle Treated Mice
b
ICT Treated Mice
c
CBLB502 Treated Mice
d
Combo Treated Mice (Flagellin i.t. + ICT)
Tlr -/- Mice
e
Vehicle Treated Mice
f
ICT Treated Mice
g
CBLB502 Treated Mice
h
Combo Treated Mice (Flagellin i.t. + ICT)
Total Photon Flux (log [photons/sec])
Tumor Volume (cm3)
Mouse 1
Mouse 2
Mouse 3
Mouse 4
Mouse 5
Mouse 6
Mouse 7
Mouse 8
Mouse 9
Mouse 10

Supplementary Figure 6. Tumor progression measurements of *Tlr5*$^{+/+}$ and *Tlr5*$^{-/-}$ BALB/c mice implanted with 4T1 *FUGW-FL* tumor cells. (a) Tumor size of vehicle-treated negative control mice *Tlr5*$^{+/+}$ (n=7) measured by bioluminescence imaging (total photon flux, left panel) and caliper measurements (tumor volume, right panel). (b) Tumor size of ICT-treated negative control mice *Tlr5*$^{+/+}$ (n=2) and (c) CBLB502-treated control mice *Tlr5*$^{+/+}$ (n=2) measured by bioluminescence imaging (total photon flux, left panel) and caliper measurements (tumor volume, right panel). (d) Tumor size of CBLB502 plus ICT-treated *Tlr*5$^{+/+}$ mice (n=10) measured by bioluminescence imaging (total photon flux, left panel) and caliper measurements (tumor volume, right panel). Mice 2 and 4, were tumor-free for at least 18 weeks post 4T1 *FUGW-FL* tumor cells implantation. (e) Tumor size of vehicle-treated negative control mice *Tlr5*$^{-/-}$ (n=7) measured by bioluminescence imaging (total photon flux, left panel) and caliper measurements (tumor volume, right panel). (f) Tumor size of ICT-treated negative control mice *Tlr5*$^{-/-}$ (n=4) and (g) CBLB502-treated negative control mice *Tlr5*$^{-/-}$ (n=5) measured by bioluminescence imaging (total photon flux, left panel) and caliper measurements (tumor volume, right panel). (h) Tumor size of CBLB502 plus ICT-treated *Tlr5*$^{-/-}$ mice (n=10) measured by bioluminescence imaging (total photon flux, left panel) and caliper measurements (tumor volume, right panel).

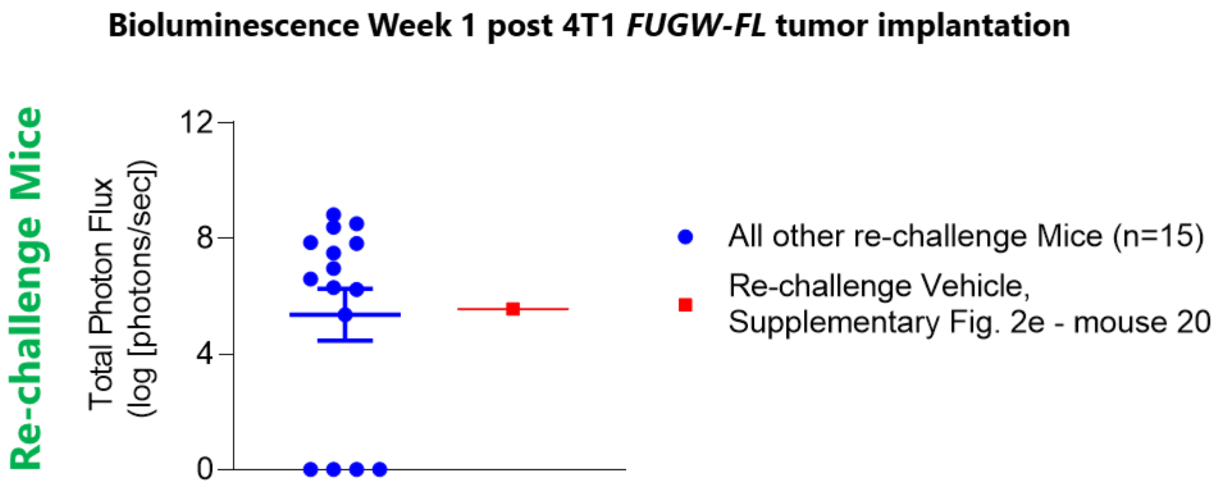

Supplementary Figure 7. Comparison of bioluminescence (total photon flux) of re-challenge vehicle control mouse (Supplementary Fig. 2e – mouse 20) and all other re-challenge mice one-week post 4T1-*FUGW-FL* tumor implantation (n=15, mean ± SEM).

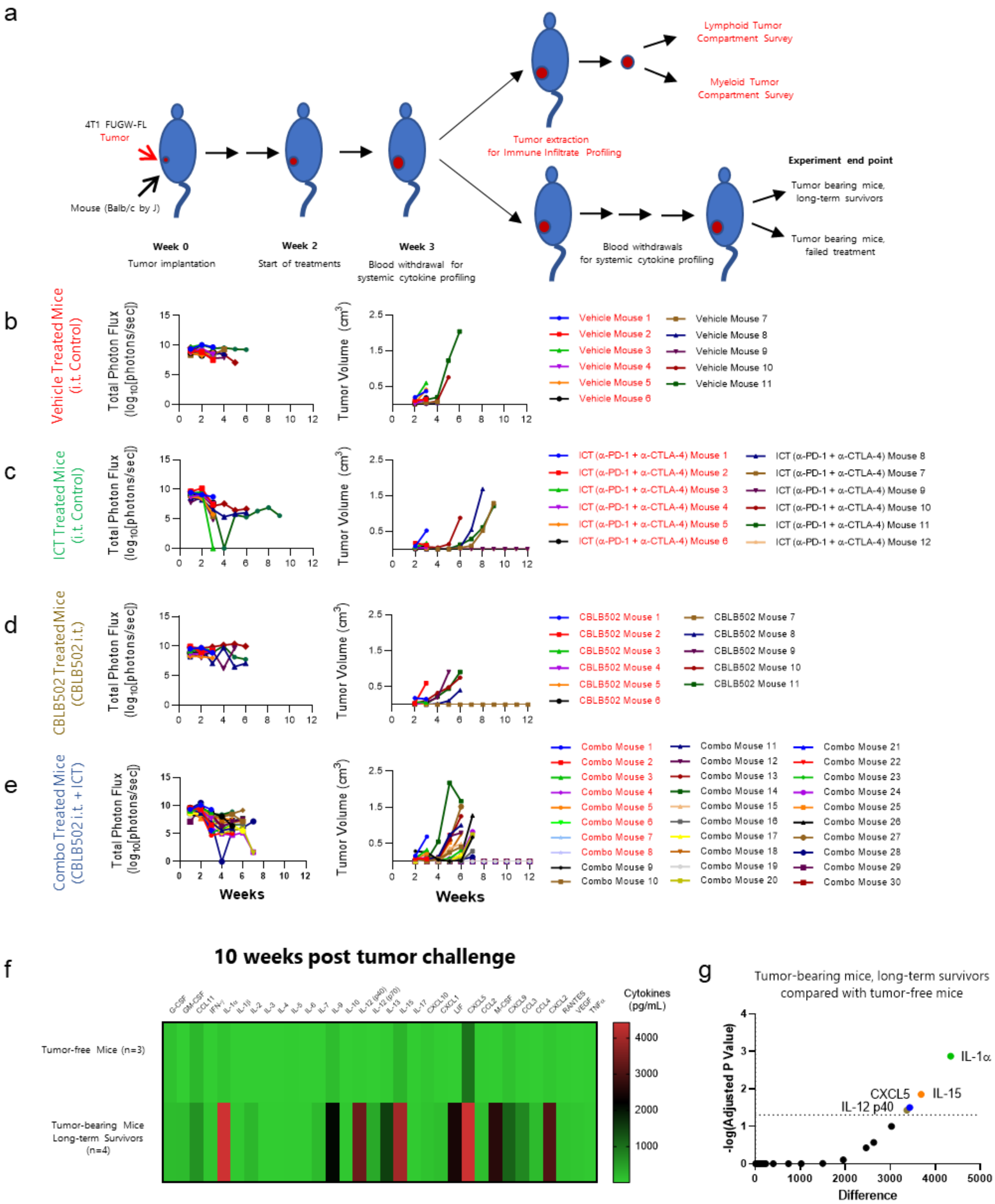

a
4T1 FUGW-FL Tumor
Mouse (Balb/c by J)
Week 0
Tumor implantation
Week 2
Start of treatments
Week 3
Blood withdrawal for systemic cytokine profiling
Tumor extraction for Immune Infiltrate Profiling
Lymphoid Tumor Compartment Survey
Myeloid Tumor Compartment Survey
Blood withdrawals for systemic cytokine profiling
Experiment end point
Tumor bearing mice, long-term survivors
Tumor bearing mice, failed treatment
b
Vehicle Treated Mice (i.t. Control)
c
ICT Treated Mice (i.t. Control)
d
CBLB502 Treated Mice (CBLB502 i.t.)
e
Combo Treated Mice (CBLB502 i.t. + ICT)
Total Photon Flux (log10[photons/sec])
Tumor Volume (cm3)
Weeks
f
10 weeks post tumor challenge
Tumor-free Mice (n=3)
Tumor-bearing Mice Long-term Survivors (n=4)
Cytokines (pg/mL)
g
Tumor-bearing mice, long-term survivors compared with tumor-free mice
-log(Adjusted P Value)
Difference
IL-1α
CXCL5
IL-15
IL-12 p40

Supplementary Figure 8. Tumor immune profile and cytokine profile experiment. (a) Model describing experiment design for cytokine and tumor immune profiling. Mice were either euthanized three weeks post 4T1 *FUGW-FL* tumor implantation (red) or allowed to complete to experiment end point (black) (12-week post tumor implantation), (b) Tumor size of vehicle-treated control mice (n=11) by bioluminescence imaging (total photon flux, left panel) and caliper measurements (tumor volume, right panel). (c) Tumor size of ICT treated mice (n=12) measured by bioluminescence imaging (total photon flux, left panel) and caliper measurements (tumor volume, right panel). (d) Tumor size of CBLB502 treated mice (n=11) measured by bioluminescence imaging (total photon flux, left panel) and caliper measurements (tumor volume, right panel). (e) Tumor size of combo treated mice (CBLB502 in combination with ICT) (n=30) measured by bioluminescence imaging (total photon flux, left panel) and caliper measurements (tumor volume, right panel). (f) Heatmap of 32 peripheral blood cytokines ten weeks post 4T1 *FUGW-FL* tumor implantation of tumor-free mice (n=3) and tumor-bearing mice (n = 4, long-term survivors). (h) Volcano plot highlighting detectable statistical differences between peripheral blood cytokines ten weeks post orthotopic tumor implantation.

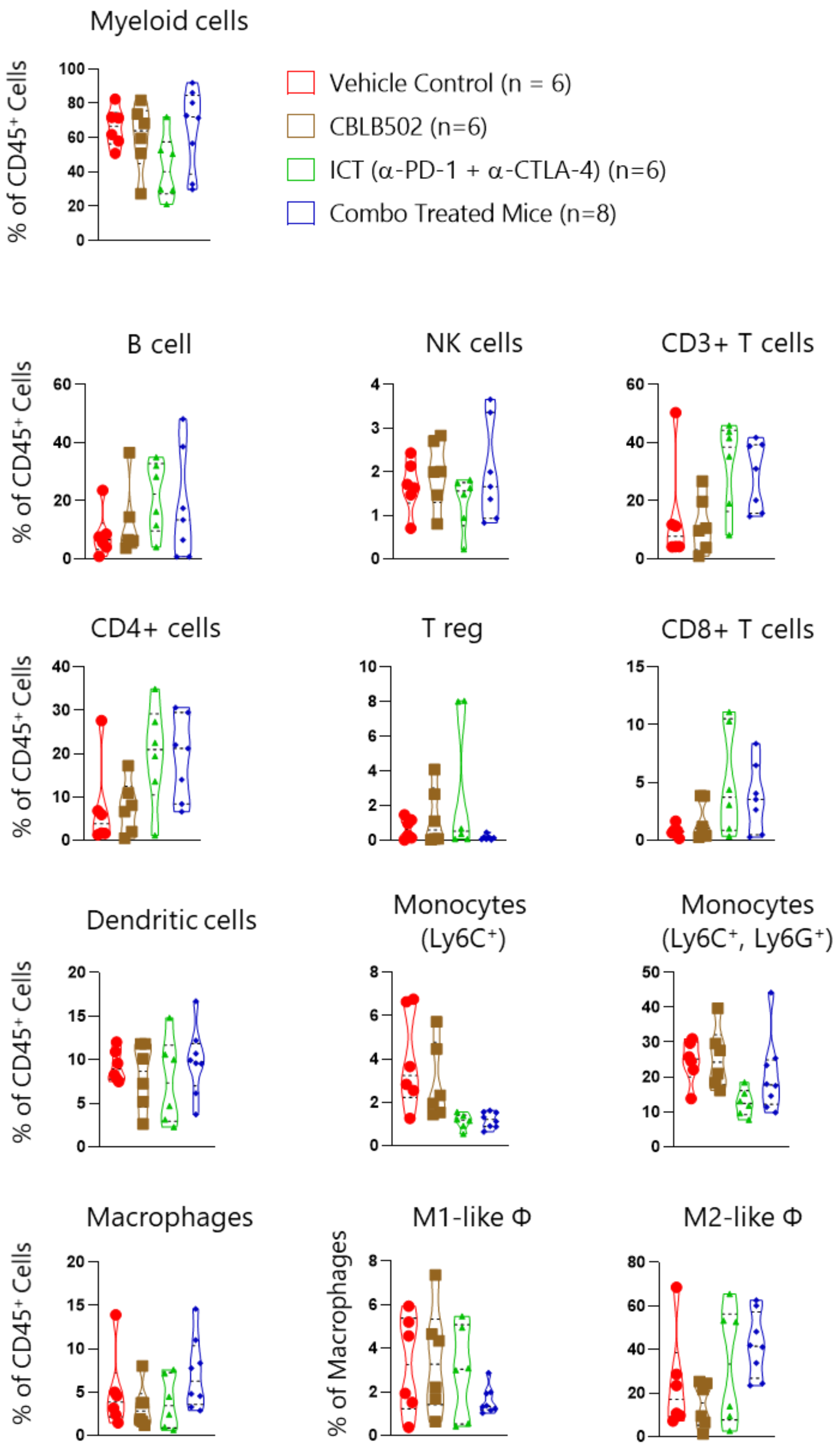

Supplementary Figure 9. Tumor immune profile of mice implanted with 4T1 *FUGW-FL* tumor cells. Myeloid and lymphoid immune cells were profiled by flow cytometry and shown as a percentage of $CD45^+$ cells of vehicle control (n=6), CBLB502 (n=6), ICT (n=6), and Combo (CBLB502 + ICT) (n=8) treated mice (Violin plot representing third quartile, median, and first quartile).

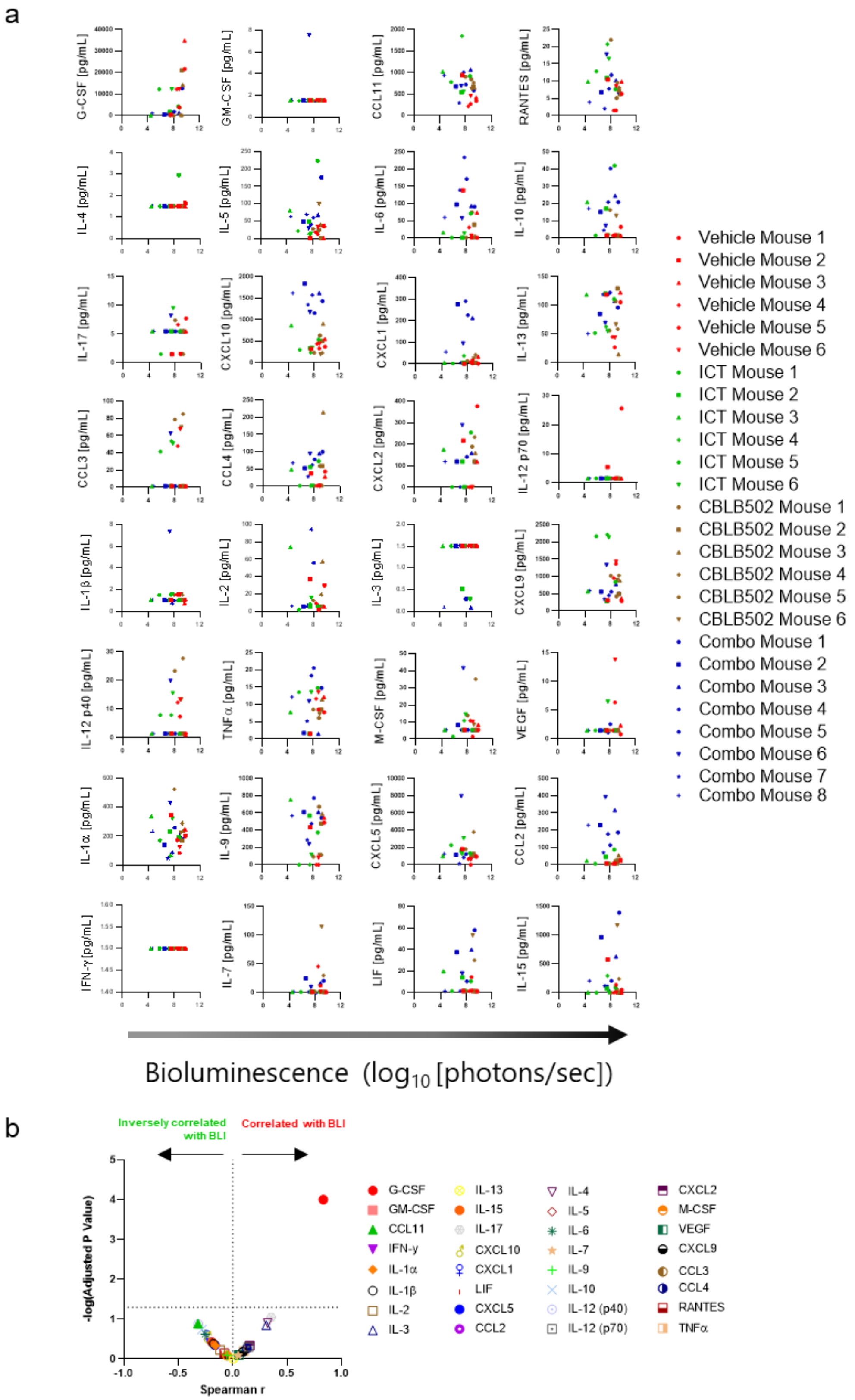
a
G-CSF [pg/mL]
GM-CSF [pg/mL]
CCL11 [pg/mL]
RANTES [pg/mL]
IL-4 [pg/mL]
IL-5 [pg/mL]
IL-6 [pg/mL]
IL-10 [pg/mL]
IL-17 [pg/mL]
CXCL10 [pg/mL]
CXCL1 [pg/mL]
IL-13 [pg/mL]
CCL3 [pg/mL]
CCL4 [pg/mL]
CXCL2 [pg/mL]
IL-12 p70 [pg/mL]
IL-1β [pg/mL]
IL-2 [pg/mL]
IL-3 [pg/mL]
CXCL9 [pg/mL]
IL-12 p40 [pg/mL]
TNFα [pg/mL]
M-CSF [pg/mL]
VEGF [pg/mL]
IL-1α [pg/mL]
IL-9 [pg/mL]
CXCL5 [pg/mL]
CCL2 [pg/mL]
IFN-γ [pg/mL]
IL-7 [pg/mL]
LIF [pg/mL]
IL-15 [pg/mL]
Vehicle Mouse 1
Vehicle Mouse 2
Vehicle Mouse 3
Vehicle Mouse 4
Vehicle Mouse 5
Vehicle Mouse 6
ICT Mouse 1
ICT Mouse 2
ICT Mouse 3
ICT Mouse 4
ICT Mouse 5
ICT Mouse 6
CBLB502 Mouse 1
CBLB502 Mouse 2
CBLB502 Mouse 3
CBLB502 Mouse 4
CBLB502 Mouse 5
CBLB502 Mouse 6
Combo Mouse 1
Combo Mouse 2
Combo Mouse 3
Combo Mouse 4
Combo Mouse 5
Combo Mouse 6
Combo Mouse 7
Combo Mouse 8
Bioluminescence (log10 [photons/sec])
b
Inversely correlated with BLI
Correlated with BLI
-log(Adjusted P Value)
Spearman r
G-CSF
GM-CSF
CCL11
IFN-γ
IL-1α
IL-1β
IL-2
IL-3
IL-13
IL-15
IL-17
CXCL10
CXCL1
LIF
CXCL5
CCL2
IL-4
IL-5
IL-6
IL-7
IL-9
IL-10
IL-12 (p40)
IL-12 (p70)
CXCL2
M-CSF
VEGF
CXCL9
CCL3
CCL4
RANTES
TNFα

Supplementary Figure 10. Blood-bourne profile of vehicle and treated mice, three weeks post orthotopic 4T1 *FUGW-FL* tumor implantation. (a) Levels of G-CSF, GM-CSF, CCL11, IFN-y, IL-1α, IL-1b, IL-2, IL-3, IL-4, IL-5, IL-6, IL-7, IL-9, IL-10, IL-12 (p40), IL-12 (p70), IL-13, IL-15, IL-17, CXCL10, CXCL1, LIF, CXCL5, CCL2, M-CSF, CXCL9, CCL3, CCL4, CXCL2, RANTES, VEGF, and TNFα of vehicle and treated mice, assigned their respective week 3 Log BLI measurements. (b) Volcano plot highlighting correlation between blood-bourne cytokine and predicted survival. Vertical line demarks negative and positive Spearman r correlation values. Line of identity indicates a $p = 0.05$).

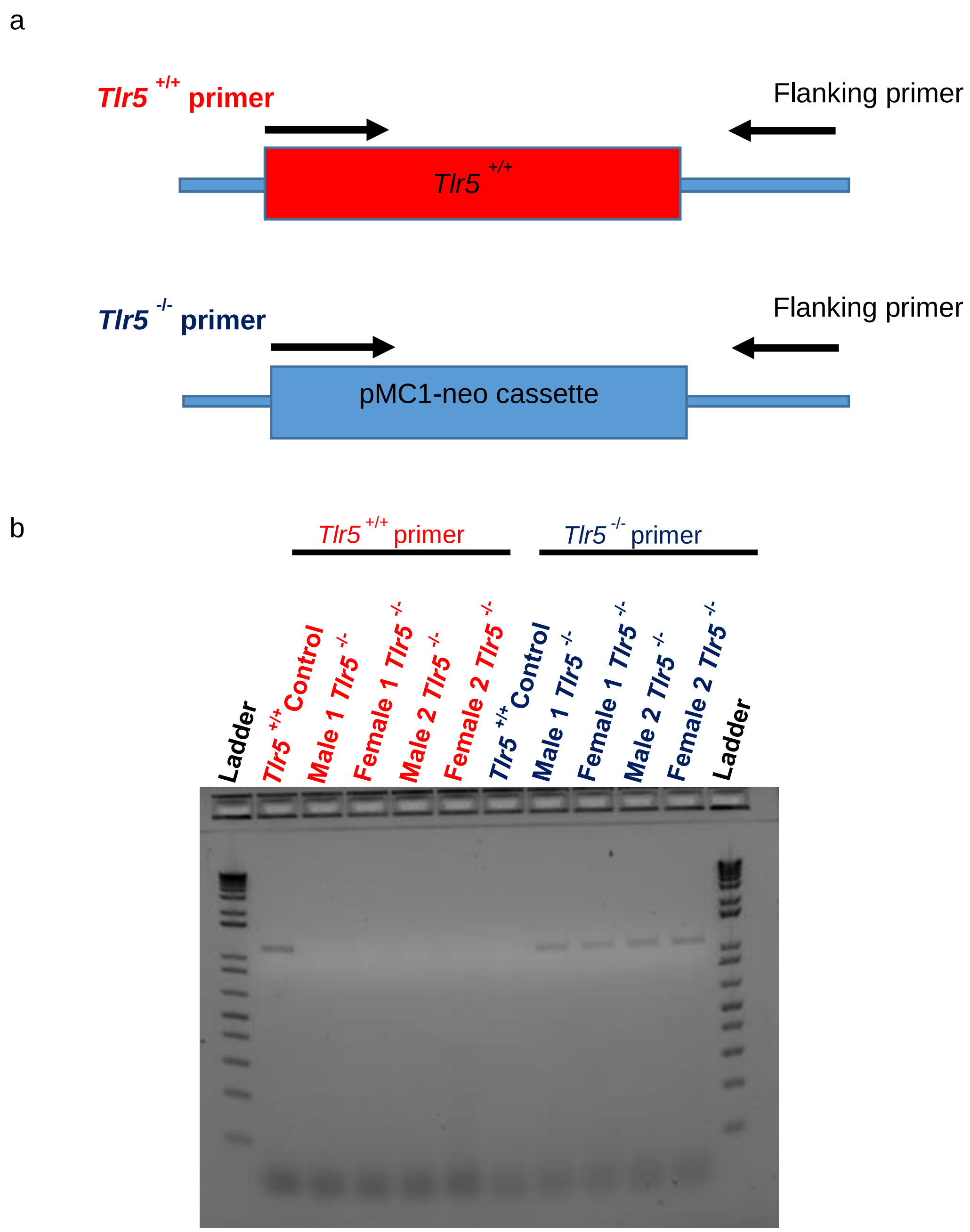

Supplementary Figure 11. PCR confirming *Tlr5*$^{+/+}$ and *Tlr5*$^{-/-}$ genotypes. A) Drawing depicting PCR amplification strategy. B) Genomic DNA was taken from *Tlr5*$^{+/+}$ and *Tlr5*$^{-/-}$ mice and *Tlr5* region was amplified using complimentary primers to confirm *Tlr5* genotype.

Supplementary Table 1: Cytokine Profile *in vitro*.

Cytokine	Vehicle (PBS)*	CBLB502 (1 µg/mL)*	Fold Difference
Axl	254	194	0.8
CXCL13	158	49	0.3
CD30 Ligand	78	43	0.6
CD30	45	26	0.6
CD40	86	37	0.4
CXCL10	116	147	1
CCL27	899	789	1
CXCL16	466	1,070	2
CCL11	30	195	7
CCL23	33	37	1
Fas Ligand	139	83	0.6
CX3CL1	2,270	1,940	0.9
G-CSF	1,670	3,580	2
GM-CSF	247	168	0.7
IFN-γ	78	67	0.9
IGFBP-3	983	1,040	1
IGFBP-5	110	370	3
IGFBP-6	194	453	2
IL-1α	880	1,010	1
IL-1β	136	253	2
IL-2	106	245	2
IL-3	64	258	4
IL-3 RB	37	257	7
IL-4	905	499	0.6
IL-5	126	63	0.5
IL-6	144	42	0.3
IL-9	227	150	0.7
IL-10	41	77	2
IL-12 p40/p70	57	278	5
IL-12 p70	746	1,060	1
IL-13	178	539	3
IL-17A	255	464	2
CXCL1	4,100	9,030	2
Leptin R	154	584	4
Leptin	102	751	7
CXCL5	3,080	7,480	2
L-Selectin	9	257	29

Supplementary Table 1 Continuation			
XCL1	168	242	1
CCL2	975	5,280	5
CCL12	325	349	1
M-CSF	752	916	1
CXCL9	88	163	2
CCL3	52	185	4
CCL9	93	615	7
CXCL2	831	1,590	2
CCL19	200	527	3
CCL20	252	1,620	6
CXCL4	611	817	1
P-Selectin	508	523	1
CCL5	1,550	2,500	2
SCF	1	97	97
CXCL12	161	47	0.3
CCL17	237	195	0.8
CCL1	963	813	0.8
CCL25	170	195	1
TIMP-1	804	776	1
TNFα	93	219	2
TNF RI	191	506	3
TNF RII	237	540	2
TPO	76	232	3
VCAM-1	382	896	2
VEGF-A	12	35	3

*Normalized densitometry

Supplementary Table 2: Murine 4T1 Carcinoma Experiments.

**4T1 Murine Carcinoma Experiments**	**Innate immune activating treatment**				**ICT (i.p.)**
Experiment 1	Flagellin (i.t.)				With or without
Experiment 2	Flagellin (i.t.)	CBLB502 High Dose (i.t.)			With or without
Experiment 3	Flagellin (i.t.)	CBLB502 High Dose (i.t.)	CBLB502 Low Dose (i.t.)		With or without
Experiment 4	Flagellin (i.t.)	CBLB502 High Dose (i.t.)	CBLB502 Low Dose (i.t.)		With or without
Experiment 5			CBLB502 Low Dose (i.t.)	CBLB502 Low Dose (i.p.)	With or without
Experiment 6 (Cytokine Profile)			CBLB502 Low Dose (i.t.)	CBLB502 Low Dose (i.p.)	With or without
Experiment 7 (Re-challenge exp. & cytokine profile)	No Treatment	No Treatment	No Treatment	No Treatment	No Treatment
Experiment 8 (Cytokine and/or immune Profile)			CBLB502 Low Dose (i.t.)		With or without
Experiment 9 (Cytokine and immune Profile)			CBLB502 Low Dose (i.t.)		With or without

Intratumoral Injection (i.t.); Intraperitoneal Injection (i.p.); ICT (α-CTLA-4 + α- PD-1).

Supplementary Table 3: B16-F10 Melanoma Experiments.

**B16-F10 Melanoma Experiments**	**Innate immune activating treatment (i.t)**	**Immune checkpoint therapy control (i.p.)**	**Combination treatment**
Experiment 1	N/A	N/A	CBLB502 (i.t.) + ICT (i.p)
Experiment 2	CBLB502 Low Dose (i.t.)	α-CTLA-4 + α-PD-1	CBLB502 (i.t.) + ICT (i.p)
Experiment 3	CBLB502 Low Dose (i.t.)	α-CTLA-4 + α-PD-1	CBLB502 (i.t.) + ICT (i.p)
Experiment 4	CBLB502 Low Dose (i.t.)	α-CTLA-4 + α-PD-1	CBLB502 (i.t.) + ICT (i.p)

Intratumoral Injection (i.t.); Intraperitoneal Injection (i.p.); ICT (α-CTLA-4 + α- PD-1).

Supplementary Table 4: *Tlr5* Knockout Mice Experiment.

***Tlr5* Status**	**Mouse Age (weeks)**	**Vehicle Control (i.t. and i.p)**	**Immune checkpoint therapy control (i.p.)**	**Innate immune activating treatment control (i.t)**
Tlr5 $^{+/+}$	9 to 11	PBS	N/A	N/A
Tlr5 $^{+/+}$	9 to 11	N/A	α-CTLA-4 + α-PD-1	CBLB502
Tlr5 $^{-/-}$	10 to 13	PBS	N/A	N/A
Tlr5 $^{-/-}$	10 to 13	N/A	α-CTLA-4 + α-PD-1	CBLB502

Supplementary Table 5: Re-challenge Experiment.

Original cohort	Mouse I.D.	Time Alive Post- tumor Implantation (weeks)	Outcome (Post-re-challenge)
Tumor-Naïve	Fig. 3b-Naïve Mouse 1	51*	Dead W6
Tumor-Naïve	Fig. 3b -Naïve Mouse 2	51*	Dead W6
Tumor-Naïve	Fig. 3b -Naïve Mouse 3	51*	Dead W6
Tumor-Naïve	Fig. 3b-Naïve Mouse 4	51*	Dead W5
Tumor-Naïve	Fig. 3b-Naïve Mouse 5	51*	Dead W5
Tumor-Naïve	Fig. 3b-Naïve Mouse 6	22*	Dead W6
Tumor-Naïve	Fig. 3b-Naïve Mouse 7	22*	Dead W6
Tumor-Naïve	Fig. 3b-Naïve Mouse 8	22*	Dead W4
Tumor-Naïve	Fig. 3b-Naïve Mouse 9	22*	Dead W6
Tumor-Naïve	Fig. 3b-Naïve Mouse 10	22*	Dead W6
Tumor-Naïve	Fig. 3b-Naïve Mouse 11	18*	Dead W6
Tumor-Naïve	Fig. 3b-Naïve Mouse 12	18*	Dead W6
Tumor-Naïve	Fig. 3b-Naïve Mouse 13	18*	Dead W6
Tumor-Naïve	Fig. 3b-Naïve Mouse 14	18*	Dead W6
Vehicle	Supp. Fig. 2e – Mouse 20	18	Died during procedure
Flagellin (i.t.)	Supp. Fig. 2c – Mouse 13	40	Alive
CBLB502 10µg (i.t.)	Supp. Fig. 3d – Mouse 5	51	Dead W7
ICT	Supp. Fig. 2f – Mouse 23	22	Dead W6
ICT	Supp. Fig. 4c – Mouse 3	18	Alive
Flagellin (i.t.) + ICT	Supp. Fig. 2d – Mouse 2	54	Alive
Flagellin (i.t.) + ICT	Supp. Fig. 2d – Mouse 6	51	Alive
Flagellin (i.t.) + ICT	Supp. Fig. 2d – Mouse 7	51	Alive
CBLB502 1µg (i.t.) + ICT	Supp. Fig. 2h – Mouse 1	40	Dead W6
CBLB502 1µg (i.t.) + ICT	Supp. Fig. 2h – Mouse 4	40	Alive
CBLB502 1µg (i.t.) + ICT	Supp. Fig. 2h – Mouse 10	38	Alive
CBLB502 1µg (i.t.) + ICT	Supp. Fig. 2h – Mouse 19	22	Alive
CBLB502 1µg (i.t.) + ICT	Supp. Fig. 2h – Mouse 22	18	Alive
CBLB502 1µg (i.t.) + ICT	Supp. Fig. 2h – Mouse 26	18	Alive
CBLB502 1µg (i.p.) + ICT	Supp. Fig. 4e – Mouse 1	22	Alive
CBLB502 1µg (i.p.) + ICT	Supp. Fig. 4e – Mouse 5	22	Alive

*Tumor-naïve mice are age-matched mice that were part of the original cohort of mice, but were not implanted with tumor cells during the original experiments.

Supplementary Table 6: Week 3 cytokine profile *in vivo*.

	Healthy Tumor-free Mice (pg/mL)	Vehicle Control (pg/mL)	Fold Change (Vehicle/Tumor-free Mice)	Failed Treatment (pg/mL)	Fold Change (Failed/Vehicle)	Long-term Survivors (pg/mL)	Fold Change (Survivor/Failed)
G-CSF	300	8,100	30	2,500	0.3	2300	0.9
GM-CSF	1.5	1.5	1	2	1.3	2	1
CCL11	730	500	0.7	900	1.8	700	0.8
IFN-γ	1.5	3	2	3	1	2	0.7
IL-1α	850	840	1	570	0.7	250	0.4
IL-1β	65	2	0.03	2	1	2	1
IL-2	6	3	0.5	19	6.3	8	0.4
IL-3	3	1.5	0.5	1	0.7	2	2
IL-4	33	1.5	0.1	2	1.3	2	1
IL-5	40	8	0.2	50	6.3	30	0.6
IL-6	150	6	0.04	80	13	13	0.2
IL-7	10	3	0.3	20	7	300	15
IL-9	60	40	0.7	160	4	80	0.5
IL-10	190	2	0.01	9	4.5	4	0.4
IL-12 (p40)	12	6	0.5	12	2	40	3
IL-12 (p70)	300	1.5	0.01	2	1	2	1
IL-13	360	20	0.1	60	3	70	1.2
IL-15	360	1.5	0.004	220	150	2300	11
IL-17	160	3	0.02	4	1.4	3	0.9
CXCL10	160	240	1.5	800	3.3	260	0.3
CXCL1	37	22	0.6	94	4.2	14	0.2
LIF	1.5	1.5	1	20	13	90	4.5
**CXCL5**	**6600**	**7500**	**1.1**	**5000**	**0.7**	**1200**	**0.2**
CCL2	160	4	0.03	120	30	6	0.1
M-CSF	7	4	0.6	15	3.8	30	2
CXCL9	400	440	1.1	1200	2.7	1700	1.4
CCL3	33	34	1	40	1.2	60	1.5
CCL4	6	20	3	40	2	2	0.04
CXCL2	24	1.5	0.06	120	80	220	1.8
CCL5	50	12	0.2	10	0.8	11	1.1
VEGF	1.5	1.5	1	1	0.7	3	3
TNFα	90	5	0.1	7	1.4	7	1

Healthy Tumor-free Mice (n = 8), vehicle control mice (n = 11), failed treatment (n =26), and long-term survivors (n = 4).

Supplementary Table 7: Week 5-7 cytokine profile *in vivo.*

	Healthy Tumor-free Mice (pg/mL)	Vehicle Control (pg/mL)	Fold Change (Vehicle/Tumor-free Mice)	Failed Treatment (pg/mL)	Fold Change (Failed/Vehicle)	Long-term Survivors (pg/mL)	Fold Change (Survivor/Failed)
**G-CSF**	**530**	**11,000**	**21**	**13,000**	**1**	**180**	**0.01**
GM-CSF	3	1	0.3	8	8	91	11
CCL11	1,000	460	0.5	560	1	700	1
IL-13	27	12	0.4	48	4	400	8.3
IL-1α	1,700	840	0.5	2,700	3	3,200	1.2
IL-1β	160	2	0.01	33	17	43	1.3
IL-2	6	2	0.3	8	4	121	15
IL-3	6	1.6	0.3	1.4	1	1.6	1.1
IL-4	81	0.5	0.01	2	4	1.4	0.7
IL-5	90	6	0.07	13	2	5	0.4
IL-6	560	1	0.002	51	51	28	0.6
IL-7	1.6	160	100	330	2	450	1.4
IL-9	370	350	1	250	1	790	3.2
IFN-γ	1	2.5	2.5	4.5	2	33	7.3
IL-10	770	1.4	0.002	68	49	33	0.5
IL-12 p40	7	6.6	0.9	2,000	303	3,300	1.7
IL-12 p70	1,100	43	0.04	83	2	71	0.9
LIF	1.6	23	14	61	3	126	2.1
CXCL5	14,000	13,000	0.9	13,000	1	10,000	0.8
**IL-15**	**144**	**790**	**5.5**	**4,400**	**6**	**7,800**	**1.8**
IL-17	30	1.7	0.06	2.2	1	1.4	0.6
CXCL10	200	360	1.8	450	1	180	0.4
CXCL1	79	27	0.3	39	1	170	4.4
CCL2	560	12	0.02	50	4	63	1.3
CCL3	36	61	1.1	59	1	240	4.1
CCL4	36	61	1.7	50	1	74	1.5
M-CSF	16	3	0.2	1,600	533	2,800	1.8
CXCL2	170	140	0.8	1,800	13	2,700	1.5
CXCL9	180	410	2.3	450	1	450	1
CCL5	110	11	0.1	19	2	22	1.2
VEGF	1	1	1	3	3	18	6
TNF-α	320	4	0.01	7	2	5	0.7

Cytokines highlighted in **Bold** showed statistically significant differences: G-CSF, Naïve Tumor-free Mice compared with Vehicle Control ($p < 0.0001$, two-way ANOVA, Turkey's multiple comparison test and $p < 0.0001$, t-test); G-CSF, Vehicle Control compared with Long-term Survivors ($p < 0.0001$, two-way ANOVA, Turkey's multiple comparison test and $p < 0.0001$, t-

test); G-CSF, Failed Treatment compared with Long-term Survivors ($p < 0.0001$, two-way ANOVA, Turkey's multiple comparison test and $p < 0.0001$, t-test); Of note there was no detectable significant difference in: Vehicle Control compared with Failed Treatment ($p < 0.3$, two-way ANOVA, Turkey's multiple comparison test and $p < 0.1$, t-test) and Naïve Tumor-free Mice compared with Long-term Survivors ($p < 0.9$, two-way ANOVA, Turkey's multiple comparison test and $p < 0.8$, t-test). IL-15, Vehicle Control compared with Long-term Survivors ($p < 0.0001$, two-way ANOVA, Turkey's multiple comparison test and $p < 0.0001$, t-test); IL-15, Failed Treatment compared with Long-term Survivors ($p < 0.0001$, two-way ANOVA, Turkey's multiple comparison test and $p < 0.0001$, t-test); IL-15, Naïve Tumor-free Mice compared with Long-term survivor ($p < 0.0001$, two-way ANOVA, Turkey's multiple comparison test and $p < 0.0001$, t-test).

Supplementary Table 8: Re-challenge experiment cytokine profile *in vivo*.

Cytokine	Tumor-naïve, tumor bearing (pg/mL)	Tumor survivors, re-challenge failure (pg/mL)	Fold-change re-challenged failure	Tumor survivors, re-challenge survivors (pg/mL)	Fold-change re-challenge survivors
G-CSF	10,000	10,000	1.0	540	0.1
GM-CSF	490	400	0.8	1,100	2
CCL11	44	130	3	1,200	27
IL-13	16	22	1	850	53
IL-1α	870	3,400	4	19,000	22
IL-1β	58	170	3	360	6
IL-2	7	46	7	1,800	260
IL-3	20	7	0.4	14	0.7
IL-4	2	41	21	81	41
IL-5	98	30	0.3	50	0.5
IL-6	65	44	0.7	120	2
IL-7	3	310	100	11,000	3,700
IL-9	460	1,900	4	3,400	7
IFN-γ	110	130	1	930	9
IL-10	38	2,100	55	8,000	210
IL-12 p40	450	200	0.4	1,000	2
IL-12 p70	5	51	10	1,200	240
LIF	38	180	5	16,000	420
CXCL5	11,000	11,000	1.0	8,900	0.8
**IL-15**	**34**	**5,800**	**170**	**650,000**	**19,000**
IL-17	34	52	2	32	0.9
CXCL10	450	140	0.3	210	0.5
CXCL1	31	77	3	3,800	120
CCL2	120	240	2	260	2
CCL3	92	240	3	1,800	20
CCL4	38	94	3	2,200	58
M-CSF	47	1,900	40	2,900	62
CXCL2	260	2,200	8	4,500	17
CXCL9	220	250	1	2,600	12
CCL5	38	45	1	120	3
VEGF	1	3	3	620	620
TNF-α	35	16	0.5	62	2

Cytokine highlighted in **Bold** showed detectable statistical difference: IL-15, Tumor-naïve, tumor bearing mice compared with tumor survivors, re-challenge survivors ($p < 0.0001$, two-way ANOVA, Turkey's multiple comparison test and $p < 0.0001$, t-test); IL-15, tumor survivors, re-challenge failure mice compared with tumor survivors, re-challenge survivors mice ($p < 0.0001$,

two-way ANOVA, Turkey's multiple comparison test and $p < 0.0001$, t-test). Of note there was a detectable statistical difference in IL-15 profile in tumor-naïve, tumor bearing mice compared with tumor survivors, re-challenge failure ($p < 0.0001$, t-test) but not in two-way ANOVA, Turkey's multiple comparison test, $p > 0.9$).

Supplementary Table 9: Cytokine and immune profiled mice.

Treatment*	Experiment (Supp. Table 2)	Mouse ID	Experimental Path	Lymphoid & Myeloid Panel
Vehicle	Exp. 8	Supp. Fig. 8b Mouse 1	Immune Profile & Cytokine Profiles	A
Vehicle	Exp. 8	Supp. Fig. 8b Mouse 2	Immune Profile & Cytokine Profiles	A
Vehicle	Exp. 8	Supp. Fig. 8b Mouse 3	Immune Profile & Cytokine Profiles	A
Vehicle	Exp. 9	Supp. Fig. 8b Mouse 4	Immune Profile & Cytokine Profiles	B
Vehicle	Exp. 9	Supp. Fig. 8b Mouse 5	Immune Profile & Cytokine Profiles	B
Vehicle	Exp. 9	Supp. Fig. 8b Mouse 6	Immune Profile & Cytokine Profiles	B
Vehicle	Exp. 8	Supp. Fig. 8b Mouse 7	Cytokine Profile Only	N/A
Vehicle	Exp. 8	Supp. Fig. 8b Mouse 8	Cytokine Profile Only	N/A
Vehicle	Exp. 8	Supp. Fig. 8b Mouse 9	Cytokine Profile Only	N/A
Vehicle	Exp. 6	Supp. Fig. 8b Mouse 10	Cytokine Profile Only	N/A
Vehicle	Exp. 6	Supp. Fig. 8b Mouse 11	Cytokine Profile Only	N/A
ICT	Exp. 8	Supp. Fig. 8c Mouse 1	Immune Profile & Cytokine Profiles	A
ICT	Exp. 8	Supp. Fig. 8c Mouse 2	Immune Profile & Cytokine Profiles	A
ICT	Exp. 8	Supp. Fig. 8c Mouse 3	Immune Profile & Cytokine Profiles	A
ICT	Exp. 9	Supp. Fig. 8c Mouse 4	Immune Profile & Cytokine Profiles	B
ICT	Exp. 9	Supp. Fig. 8c Mouse 5	Immune Profile & Cytokine Profiles	B
ICT	Exp. 9	Supp. Fig. 8c Mouse 6	Immune Profile & Cytokine Profiles	B
ICT	Exp. 8	Supp. Fig. 8c Mouse 7	Cytokine Profile Only	N/A
ICT	Exp. 8	Supp. Fig. 8c Mouse 8	Cytokine Profile Only	N/A
ICT	Exp. 8	Supp. Fig. 8c Mouse 9	Cytokine Profile Only	N/A
ICT	Exp. 6	Supp. Fig. 8c Mouse 10	Cytokine Profile Only	N/A
ICT	Exp. 6	Supp. Fig. 8c Mouse 11	Cytokine Profile Only	N/A
ICT	Exp. 6	Supp. Fig. 8c Mouse 12	Cytokine Profile Only	N/A
CBLB502	Exp. 8	Supp. Fig. 8d Mouse 1	Immune Profile & Cytokine Profiles	A
CBLB502	Exp. 8	Supp. Fig. 8d Mouse 2	Immune Profile & Cytokine Profiles	A
CBLB502	Exp. 8	Supp. Fig. 8d Mouse 3	Immune Profile & Cytokine Profiles	A
CBLB502	Exp. 9	Supp. Fig. 8d Mouse 4	Immune Profile & Cytokine Profiles	B
CBLB502	Exp. 9	Supp. Fig. 8d Mouse 5	Immune Profile & Cytokine Profiles	B
CBLB502	Exp. 9	Supp. Fig. 8d Mouse 6	Immune Profile & Cytokine Profiles	B
CBLB502	Exp. 8	Supp. Fig. 8d Mouse 7	Cytokine Profile Only	N/A
CBLB502	Exp. 8	Supp. Fig. 8d Mouse 8	Cytokine Profile Only	N/A
CBLB502	Exp. 8	Supp. Fig. 8d Mouse 9	Cytokine Profile Only	N/A
CBLB502	Exp. 6	Supp. Fig. 8d Mouse 10	Cytokine Profile Only	N/A
CBLB502	Exp. 6	Supp. Fig. 8d Mouse 11	Cytokine Profile Only	N/A
Combo	Exp. 8	Supp. Fig. 8e Mouse 1	Immune Profile & Cytokine Profiles	A

Treatment	Experiment (Supp. Table 2)	Mouse ID	Experimental Path	Lymphoid & Myeloid Panel
Combo	Exp. 8	Supp. Fig. 8e Mouse 2	Immune Profile & Cytokine Profiles	A
Combo	Exp. 8	Supp. Fig. 8e Mouse 3	Immune Profile & Cytokine Profiles	A
Combo	Exp. 8	Supp. Fig. 8e Mouse 4	Immune Profile & Cytokine Profiles	A
Combo	Exp. 8	Supp. Fig. 8e Mouse 5	Immune Profile & Cytokine Profiles	A
Combo	Exp. 8	Supp. Fig. 8e Mouse 6	Immune Profile & Cytokine Profiles	A
Combo	Exp. 8	Supp. Fig. 8e Mouse 7	Immune Profile & Cytokine Profiles	A
Combo	Exp. 8	Supp. Fig. 8e Mouse 8	Immune Profile & Cytokine Profiles	A
Combo	Exp. 8	Supp. Fig. 8e Mouse 9	Cytokine Profile Only	N/A
Combo	Exp. 8	Supp. Fig. 8e Mouse 10	Cytokine Profile Only	N/A
Combo	Exp. 8	Supp. Fig. 8e Mouse 11	Cytokine Profile Only	N/A
Combo	Exp. 8	Supp. Fig. 8e Mouse 12	Cytokine Profile Only	N/A
Combo	Exp. 8	Supp. Fig. 8e Mouse 13	Cytokine Profile Only	N/A
Combo	Exp. 8	Supp. Fig. 8e Mouse 14	Cytokine Profile Only	N/A
Combo	Exp. 8	Supp. Fig. 8e Mouse 15	Cytokine Profile Only	N/A
Combo	Exp. 8	Supp. Fig. 8e Mouse 16	Cytokine Profile Only	N/A
Combo	Exp. 9	Supp. Fig. 8e Mouse 17	Cytokine Profile Only	N/A
Combo	Exp. 9	Supp. Fig. 8e Mouse 18	Cytokine Profile Only	N/A
Combo	Exp. 9	Supp. Fig. 8e Mouse 19	Cytokine Profile Only	N/A
Combo	Exp. 9	Supp. Fig. 8e Mouse 20	Cytokine Profile Only	N/A
Combo	Exp. 9	Supp. Fig. 8e Mouse 21	Cytokine Profile Only	N/A
Combo	Exp. 9	Supp. Fig. 8e Mouse 22	Cytokine Profile Only	N/A
Combo	Exp. 9	Supp. Fig. 8e Mouse 23	Cytokine Profile Only	N/A
Combo	Exp. 9	Supp. Fig. 8e Mouse 24	Cytokine Profile Only	N/A
Combo	Exp. 9	Supp. Fig. 8e Mouse 25	Cytokine Profile Only	N/A
Combo	Exp. 9	Supp. Fig. 8e Mouse 26	Cytokine Profile Only	N/A
Combo	Exp. 9	Supp. Fig. 8e Mouse 27	Cytokine Profile Only	N/A
Combo	Exp. 6	Supp. Fig. 8e Mouse 28	Cytokine Profile Only	N/A
Combo	Exp. 6	Supp. Fig. 8e Mouse 29	Cytokine Profile Only	N/A
Combo	Exp. 6	Supp. Fig. 8e Mouse 30	Cytokine Profile Only	N/A

* ICT (α-CTLA-4 + α-PD-1); Combo (CBLB502 + ICT)

Supplementary Table 10: Lymphoid and Myeloid staining antibodies for flow cytometry analysis – Panel A.

Panel A	Marker	Color/Format	Target	Clone
Lymphoid Panel	Zombie UV	Zombie UV		
Lymphoid Panel	4T1 *FUGW-FL*	FITC		
Lymphoid Panel	CD285	PE	anti-mouse	ACT5
Lymphoid Panel	CD19	PE-Cy5	anti-mouse	6D5
Lymphoid Panel	CD4	PerCP-Cy5.5	anti-mouse	GK1.5
Lymphoid Panel	CD45	Alexa 700	anti-mouse	30-F11
Lymphoid Panel	Foxp3	Alexa 647	anti-mouse/human	3G3
Lymphoid Panel	CD49b	PE-dazzle 594	anti-mouse	DX5
Lymphoid Panel	CD3 epsilon	BV650	anti-mouse	17A2
Lymphoid Panel	CD8a	BV510	anti-mouse	53-6.7
Myeloid Panel	Zombie UV	Zombie UV		
Myeloid Panel	4T1 *FUGW-FL*	FITC		
Myeloid Panel	CD285	PE	anti-mouse	ACT5
Myeloid Panel	Ly6C	Brilliant Violet 510	anti-mouse	HK1.4
Myeloid Panel	CD11c	Brilliant Violet 711	anti-mouse	N418
Myeloid Panel	CD45	Brilliant Violet 650	anti-mouse	30-F11
Myeloid Panel	CD11b	PE-Cy7	anti-mouse	M1/70
Myeloid Panel	Ly6G	APC-Fire 750	anti-mouse	1A8
Myeloid Panel	CD163	PerCP-eFluor710	anti-mouse	TNKUPJ
Myeloid Panel	CD68	eFluor660	anti-mouse	FA-11
Myeloid Panel	F4/80	PE-dazzle 594	anti-mouse	BM8
Myeloid Panel	CD80	BV650	anti-mouse	16-10A1

Supplementary Table 11: Lymphoid and Myeloid staining antibodies for flow cytometry analysis – Panel B.

Panel B	Marker	Color/Format	Target	Clone
Lymphoid Panel	Zombie UV			
Lymphoid Panel	4T1 *FUGW-FL*	FITC		
Lymphoid Panel	CD285	PE	anti-mouse	ACT5
Lymphoid Panel	CD45	Alexa Fluor 700	anti-mouse	30-F11
Lymphoid Panel	CD3	Brilliant Violet 510	anti-mouse	17A2
Lymphoid Panel	CD19	PerCP/Cy5.5	anti-mouse	1D3/CD19
Lymphoid Panel	CD4	PE/Cy7	anti-mouse	GK1.5
Lymphoid Panel	CD8	Brilliant Violet 711	anti-mouse	53-6.7
Lymphoid Panel	Foxp3	Alexa Fluor 647	anti-mouse	MF-14
Lymphoid Panel	CD49b	BV421	anti-mouse	DX5
Myeloid Panel	Zombie UV			
Myeloid Panel	4T1 *FUGW-FL*	FITC		
Myeloid Panel	CD285	PE	anti-mouse	ACT5
Myeloid Panel	CD45	Alexa Fluor 700	anti-mouse	30-F11
Myeloid Panel	CD11b	PerCP/Cyanine5.5	anti-mouse/human	M1/70
Myeloid Panel	CD11c	Brilliant Violet 510	anti-mouse	N418
Myeloid Panel	F4/80	Brilliant Violet 785	anti-mouse	BM8
Myeloid Panel	Ly6G	PE/Cy7	anti-mouse	1A8
Myeloid Panel	Ly6C	Brilliant Violet 605	anti-mouse	HK1.4
Myeloid Panel	CD80	Brilliant Violet 421	anti-mouse	16-10A
Myeloid Panel	CD163	APC	anti-mouse	S15049I

Supplementary Table 12: Antibody markers used to identify immune cells.

Immune compartment	Immune Cells	Cellular Marker
Lymphoid	B Cell	*UV Zombie$^-$; CD45$^+$; CD19$^+$; CD49b$^-$
Lymphoid	NK Cells	*UV Zombie$^-$; CD45$^+$; CD19$^-$; CD3$^-$; CD49b$^+$
Lymphoid	CD3$^+$ T Cell	*UV Zombie$^-$; CD45$^+$; CD19$^-$; CD3$^+$
Lymphoid	CD4$^+$ T cells	*UV Zombie$^-$; CD45$^+$; CD19$^-$; CD3$^+$; CD4$^+$; FOXP3$^-$
Lymphoid	T regs	*UV Zombie$^-$; CD45$^+$; CD19$^-$; CD3$^+$; CD4$^+$; FOXP3$^+$
Lymphoid	CD8$^+$ T cells	*UV Zombie$^-$; CD45$^+$; CD19$^-$; CD3$^+$; CD4$^-$; CD8$^+$
Myeloid	Myeloid Cells	*UV Zombie$^-$; CD45$^+$; CD11b$^+$
Myeloid	Dendritic Cells	*UV Zombie$^-$; CD45$^+$; CD11b$^+$; CD11c$^+$, F4/80$^-$
Myeloid	Monocyte (Ly6C$^+$)	*UV Zombie$^-$; CD45$^+$; CD11b$^+$; CD11c$^-$, F4/80$^-$; Ly6C$^+$; Ly6G$^-$
Myeloid	Monocyte (Ly6C$^+$, Ly6G$^+$)	*UV Zombie$^-$; CD45$^+$; CD11b$^+$; CD11c$^-$, F4/80$^-$; Ly6C$^+$; Ly6G$^+$
Myeloid	Macrophages	*UV Zombie$^-$; CD45$^+$; CD11b$^+$; CD11c$^-$, F4/80$^+$
Myeloid	M1- like macrophages	*UV Zombie$^-$; CD45$^+$; CD11b$^+$; CD11c$^-$, F4/80$^+$; CD80$^+$, CD163$^-$
Myeloid	M2- like macrophages	*UV Zombie$^-$; CD45$^+$; CD11b$^+$; CD11c$^-$, F4/80$^+$; CD80$^-$, CD163$^+$

[*] SSC-A/FSC-A (Cells) >FSC-H/FSC-A (Single cell gating) >UV Zombie/FSC-A (UV Zombie)

Supplementary Table 13: Cytokine profile *in vivo*: Immune infiltrate profiled vehicle control mice.

Supp. Fig. 8 Vehicle*	Mouse1 (pg/mL)	Mouse 2 (pg/mL)	Mouse 3 (pg/mL)	Mouse 4 (pg/mL)	Mouse 5 (pg/mL)	Mouse 6 (pg/mL)
G-CSF	22000	360	35000	12000	4000	12000
GM-CSF	1.5	1.5	1.5	1.5	1.5	2
CCL11	350	940	430	220	280	460
IFN-y	1.5	1.5	2	1.5	2	2
IL-1α	200	340	250	170	80	120
IL-1β	N/A	1	0.7	1.5	2	2
IL-2	30	38	6	9	2	2
IL-3	2	2	2	2	2	2
IL-4	2	2	2	2	2	2
IL-5	30	2	2	18	11	32
IL-6	2	140	70	31	2	7
IL-7	2	2	2	46	12	2
IL-9	490	430	550	2	2	80
IL-10	7	2	2	2	2	2
IL-12 (p40)	2	2	1	12	7	13
IL-12 (p70)	26	3	2	2	2	2
IL-13	100	120	120	45	27	44
IL-15	49	580	6	2	130	2
IL-17	8	2	6	7	2	2
CXCL10	370	320	550	440	330	4440
CXCL1	2	2	33	9	2	13
LIF	2	2	2	2	15	2
CXCL5	950	1800	1000	650	2	600
CCL2	28	8	23	6	6	6
M-CSF	6	6	8	11	2	10
CXCL9	290	310	360	960	1400	1400
CCL3	2	2	2	48	2	68
CCL4	40	38	30	2	2	2
CXCL2	380	220	120	2	2	2
RANTES	6	11	10	2	2	9
VEGF	0.8	2	2	2	7	14
TNFα	8	2	12	12	9	14

*Vehicle (PBS)

Supplementary Table 14: Cytokine profile *in vivo*: Immune infiltrate profiled ICT treated mice.

Supp. Fig. 8 ICT*	Mouse1 (pg/mL)	Mouse 2 (pg/mL)	Mouse 3 (pg/mL)	Mouse 4 (pg/mL)	Mouse 5 (pg/mL)	Mouse 6 (pg/mL)
G-CSF	4300	1800	200	140	12000	12000
GM-CSF	2	2	2	2	2	2
CCL11	920	550	1000	1800	780	560
IFN-y	2	2	2	2	2	2
IL-1α	190	230	330	60	170	320
IL-1β	1	1	1	2	2	2
IL-2	6	6	70	8	2	16
IL-3	0.3	0.5	2	2	2	2
IL-4	3	2	2	2	2	2
IL-5	220	48	80	12	21	13
IL-6	70	2	16	2	2	14
IL-7	2	2	2	2	2	2
IL-9	380	570	750	2	2	120
IL-10	42	17	21	2	2	2
IL-12 (p40)	2	2	2	8	8	16
IL-12 (p70)	2	2	2	2	2	2
IL-13	110	120	120	63	52	55
IL-15	87	72	6	300	2	2
IL-17	6	6	6	2	2	10
CXCL10	540	340	870	240	300	350
CXCL1	6	6	6	37	2	2
LIF	11	14	20	2	2	2
CXCL5	1300	1600	1000	1300	2300	3100
CCL2	88	46	23	6	6	6
M-CSF	6	6	6	11	2	15
CXCL9	840	290	570	2200	2200	2100
CCL3	2	2	2	54	42	50
CCL4	73	56	49	2	2	2
CXCL2	260	120	180	2	2	2
RANTES	8	11	10	21	13	17
VEGF	2	2	2	2	2	7
TNFα	15	2	8	2	14	14

* ICT (α-CTLA-4 + α-PD-1)

Supplementary Table 15: Cytokine profile *in vivo*: Immune infiltrate profiled CBLB502 treated mice.

Supp. Fig. 8 CBLB502	Mouse1 (pg/mL)	Mouse 2 (pg/mL)	Mouse 3 (pg/mL)	Mouse 4 (pg/mL)	Mouse 5 (pg/mL)	Mouse 6 (pg/mL)
G-CSF	3700	21000	14000	46	68	130
GM-CSF	2	2	2	2	2	2
CCL11	850	680	650	770	900	620
IFN-y	2	2	2	2	2	2
IL-1α	180	170	240	290	520	220
IL-1β	1	1	1	2	2	2
IL-2	6	6	58	19	12	2
IL-3	2	2	2	2	2	2
IL-4	2	2	2	2	2	2
IL-5	23	2	2	38	27	99
IL-6	75	40	5	2	2	2
IL-7	2	2	2	30	2	115
IL-9	670	480	590	110	91	112
IL-10	2	2	2	2	16	13
IL-12 (p40)	2	2	2	28	23	13
IL-12 (p70)	2	2	2	2	2	2
IL-13	107	130	15	60	57	67
IL-15	58	6	6	240	2	1170
IL-17	6	6	6	2	7	2
CXCL10	630	500	900	230	230	200
CXCL1	6	6	6	40	14	24
LIF	2	2	2	30	2	53
CXCL5	980	1200	1040	3800	1800	960
CCL2	23	23	54	6	6	6
M-CSF	6	6	6	35	14	9
CXCL9	430	500	890	1030	1020	900
CCL3	2	2	2	85	80	70
CCL4	60	59	220	2	2	2
CXCL2	190	120	160	230	2	2
RANTES	5	7	6	8	22	10
VEGF	2	2	2	2	2	2
TNFα	6	9	11	9	9	7

Supplementary Table 16: Cytokine profile *in vivo*: Immune infiltrate profiled combo treated mice.

Supp. Fig. 8 Combo*	Mouse1 (pg/mL)	Mouse 2 (pg/mL)	Mouse 3 (pg/mL)	Mouse 4 (pg/mL)	Mouse 5 (pg/mL)	Mouse 6 (pg/mL)	Mouse 7 (pg/mL)	Mouse 8 (pg/mL)
G-CSF	12700	480	1300	1580	1830	850	0.59	0.60
GM-CSF	2	2	2	2	2	8	620	1100
CCL11	590	680	1070	1010	720	690	2	2
IFN-y	2	2	2	2	2	2	300	940
IL-1α	170	140	200	83	260	430	2	2
IL-1β	1	1	1	0.7	1	7	45	230
IL-2	6	6	6	90	60	6	1	1
IL-3	2	2	0.07	2	0.3	2	6	6
IL-4	2	2	2	2	2	2	2	0.07
IL-5	180	48	70	40	60	30	2	2
IL-6	90	98	90	230	170	58	68	62
IL-7	20	25	17	2	0.02	10	140	60
IL-9	550	600	612	480	770	240	2	2
IL-10	21	15	24	21	40	7	300	570
IL-12 (p40)	2	2	2	2	2	20	5	17
IL-12 (p70)	2	2	2	2	2	2	2	2
IL-13	96	85	107	120	120	70	2	2
IL-15	1400	970	630	30	200	100	120	51
IL-17	6	6	6	6	6	8	120	200
CXCL10	1400	1840	1600	1600	1160	1200	6	6
CXCL1	6	280	212	290	230	95	1350	1600
LIF	60	38	40	2	11	18	6	55
CXCL5	1050	1200	900	840	1230	8000	2	1
CCL2	190	230	320	180	110	400	100	1250
M-CSF	6	8	6	6	6	40	70	230
CXCL9	500	560	780	450	560	1300	6	6
CCL3	2	2	2	2	2	60	340	600
CCL4	100	50	95	62	80	95	2	2
CXCL2	120	120	160	2	140	300	30	70
RANTES	7	7	10	8	12	18	2	120
VEGF	2	2	2	1	3	2	2	4
TNFα	15	2	2	18	21	11	2	2

*Combo (CBLB502 + ICT)
