# Supplementary material for: TLR5 agonists enhance anti-tumor immunity and overcome resistance to immune checkpoint therapy
Source: Commun Biol. 2023 Jan 12;6:31. doi: 10.1038/s42003-022-04403-8 (PMC9837180; doi:10.1038/s42003-022-04403-8)
Supplement: Supplementary file 4 — Reporting Summary [file 42003_2022_4403_MOESM4_ESM.pdf]

nature portfolio

Corresponding author(s): David Piwnica-Worms
Last updated by author(s): Dec 7, 2022

Reporting Summary

Nature Portfolio wishes to improve the reproducibility of the work that we publish. This form provides structure for consistency and transparency in reporting. For further information on Nature Portfolio policies, see our Editorial Policies and the Editorial Policy Checklist.

Statistics

For all statistical analyses, confirm that the following items are present in the figure legend, table legend, main text, or Methods section.

n/a	Confirmed	
☐	☒	The exact sample size (n) for each experimental group/condition, given as a discrete number and unit of measurement
☐	☒	A statement on whether measurements were taken from distinct samples or whether the same sample was measured repeatedly
☐	☒	The statistical test(s) used AND whether they are one- or two-sided<br>*Only common tests should be described solely by name; describe more complex techniques in the Methods section.*
☐	☒	A description of all covariates tested
☐	☒	A description of any assumptions or corrections, such as tests of normality and adjustment for multiple comparisons
☐	☒	A full description of the statistical parameters including central tendency (e.g. means) or other basic estimates (e.g. regression coefficient) AND variation (e.g. standard deviation) or associated estimates of uncertainty (e.g. confidence intervals)
☐	☒	For null hypothesis testing, the test statistic (e.g. F, t, r) with confidence intervals, effect sizes, degrees of freedom and P value noted<br>*Give P values as exact values whenever suitable.*
☒	☐	For Bayesian analysis, information on the choice of priors and Markov chain Monte Carlo settings
☒	☐	For hierarchical and complex designs, identification of the appropriate level for tests and full reporting of outcomes
☐	☒	Estimates of effect sizes (e.g. Cohen's d, Pearson's r), indicating how they were calculated

Our web collection on statistics for biologists contains articles on many of the points above.

Software and code

Policy information about availability of computer code

Data collection	No software was used for data collection.
Data analysis	GraphPad Prism (version 8.0.0 for Windows, GraphPad Software, San Diego, California), was used for statistical analysis. FlowJo (V10) was used for flow cytometry analysis.

For manuscripts utilizing custom algorithms or software that are central to the research but not yet described in published literature, software must be made available to editors and reviewers. We strongly encourage code deposition in a community repository (e.g. GitHub). See the Nature Portfolio guidelines for submitting code & software for further information.

Data

Policy information about availability of data

All manuscripts must include a data availability statement. This statement should provide the following information, where applicable:

- Accession codes, unique identifiers, or web links for publicly available datasets
- A description of any restrictions on data availability
- For clinical datasets or third party data, please ensure that the statement adheres to our policy

All data generated or analysed during this study are included in this published article (and its supplementary information files).

Human research participants

Policy information about studies involving human research participants and Sex and Gender in Research.

Reporting on sex and gender	N/A
Population characteristics	N/A
Recruitment	N/A
Ethics oversight	N/A

Note that full information on the approval of the study protocol must also be provided in the manuscript.

Field-specific reporting

Please select the one below that is the best fit for your research. If you are not sure, read the appropriate sections before making your selection.

☒ Life sciences ☐ Behavioural & social sciences ☐ Ecological, evolutionary & environmental sciences

For a reference copy of the document with all sections, see nature.com/documents/nr-reporting-summary-flat.pdf

Life sciences study design

All studies must disclose on these points even when the disclosure is negative.

Sample size	No sample size calculator was used to determine sample sizes. For survival studies a minimal of 3 mice per treatment group per experiment was used, each survival study was repeated at least 3 times.
Data exclusions	One vehicle-treated mouse was identified as an outlier using the ROUT Method for having low photon flux measurement at week 2 before treatment began and was omitted from the study.
Replication	All attempts at reproducibility were successful. For long-term survival animal studies (testing the effects of TLR5 agonist alone or in combination with ICT) a minimal of 4 independent experiments were performed. Each experiment had a least 3 mice per treatment group. Note: experiments are annotated in Supplemental Material section of the manuscript.<br><br>Re-challenge experiment was performed once. Two independent experiment were conducted for Tlr5 Knockout mice experiments, due to limited availability of TLR5 -/- mice. Treatment groups containing TLR5 -/- mice were assigned as follow: Vehicle n = 7, ICT only n =4, and CBLB502 n=5, CBLB502 + ICT n = 10.
Randomization	Mice were randomized two weeks post tumor implantation, using Random.org random sequence generator.
Blinding	Animal studies were not blinded during data collection. Experimental groups were treated for two weeks, while tumor volume progression were taken for each mice.

Reporting for specific materials, systems and methods

We require information from authors about some types of materials, experimental systems and methods used in many studies. Here, indicate whether each material, system or method listed is relevant to your study. If you are not sure if a list item applies to your research, read the appropriate section before selecting a response.

Materials & experimental systems

n/a	Involved in the study	
☐	☒	Antibodies
☐	☒	Eukaryotic cell lines
☒	☐	Palaeontology and archaeology
☐	☒	Animals and other organisms
☒	☐	Clinical data
☒	☐	Dual use research of concern

Methods

n/a	Involved in the study	
☐	☐	ChIP-seq
☐	☒	Flow cytometry
☐	☐	MRI-based neuroimaging

Antibodies

Antibodies used

Flow cytometry

Marker / Color/Format / Host/Target / Isotype / Clone / / Catalog

Ly-6C / Brilliant Violet 510 / Rat anti-Mouse / IgG2c κ / HK1.4 / Invivogen / 128033
CD11c / Brilliant Violet 711 / Hamster anti-Mouse / IgG / N418 / Invivogen / 117349
CD45 / Brilliant Violet 650 / Rat anti-Mouse / IgG2b κ / 30-F11 / Invivogen / 103151
CD11b / PE-Cy7 / Rat anti-Mouse / IgG2b κ / M1/70 / Invivogen / 101215
Ly-6G / APC-Fire 750 / Rat anti-Mouse / IgG2a κ / 1A8 / Invivogen / 127651
F4/80 / PE-dazzle 594 / Rat anti-Mouse / IgG2a κ / BM8 / Invivogen / 123145
TLR5-CD285 / PE / anti- Mouse / IgG1 κ / ACT5 / Invivogen / 565645
CD80 / BV650 / Hamster anti-Mouse / IgG / 16-10A1 / Invivogen / 104731
CD163 / PerCP-eFluor710 / Rat anti-Mouse / IgG2a κ / TNKUPJ / Thermo Fisher / 46-1631-82
CD68 / eFluor660 / Rat anti-Mouse / IgG2a κ / FA-11 / Thermo Fisher / 50-0681-82
CD276 / BV421 / Rat anti-Mouse / IgG2a κ / MIH32 / BD Bioscience / 562634
CD19 / PE-Cy5 / Rat anti-Mouse / IgG2a κ / 6D5 / Invivogen / 115509
CD4 / PerCP-Cy5.5 / Rat anti-Mouse / IgG2a κ / GK1.5 / Invivogen / 100433
CD45 / Alexa 700 / Rat anti-Mouse / IgG2b κ / 30-F11 / Invivogen / 103127
CD49b pan-NK cells / PE-dazzle 594 / Rat anti-Mouse / IgM κ / DX5 / Invivogen / 108923
CD3 epsilon / BV650 / Rat anti-Mouse / IgG2a κ / 17A2 / Invivogen / 100229
CD8a / BV510 / Rat anti-Mouse / IgG2a, k / 53-6.7 / Invivogen / 100751
OX40 / APC-Fire780 / Rat anti-Mouse / IgG1 κ / OX-86 / Invivogen / 119422
ICOS / BV605 / AH anti Human, Mouse / Armenian Hamster IgG / C398.4A / Invivogen / 313537
PD-1 / BV711 / anti- Mouse / IgG2a, k / 29F.1A12 / Invivogen / 135231
LAG-3 / BV785 / anti- Mouse / IgG1 κ / C9B7W / Invivogen / 125219
CTLA-4 / PE-Cy7 / anti- Mouse / Armenian Hamster IgG / UC10-4B9 / Invivogen / 106313
Foxp3 / Alexa 647 / Human, Mouse / IgG1 κ / 3G3 / Thermo Fisher / MA5-18160
CD276 / BV421 / anti- Mouse / IgG2a, k / MIH32 / BD Bioscience / 562634

Immune Checkpoint therapy

InVivoPlus anti-mouse CTLA-4 (CD152)
Clone 9D9
Vendor BioXCell
Catalog # BP0164

InVivoMAb anti-mouse PD-1 (CD279)
Clone RMP1-14
Vendor BioXCell
Catalog # BP0146

In Cellulo Cytokine Profile

Mouse Cytokine Array C1000
Vendor: Ray Biotech
Product code: AAM-CYT-1000

Mice Cytokine Assay was conducted by Baylor College of Medicine, (Houston, TX) Proteomics Core.
Platform Luminex
MILLIPLEX Mouse 32-Plex Cytokine Panel 1

Validation

Describe the validation of each primary antibody for the species and application, noting any validation statements on the manufacturer's website, relevant citations, antibody profiles in online databases, or data provided in the manuscript.

Eukaryotic cell lines

Policy information about cell lines and Sex and Gender in Research

Cell line source(s)	B16-F10 cells were obtained from the MD Anderson Cell Line Core<br>4T1 cells were obtained from ATCC
Authentication	Respective depository certification.
Mycoplasma contamination	Cells tested negative for Mycoplasma.

Commonly misidentified lines
(See ICLAC register)

N/A

Animals and other research organisms

Policy information about studies involving animals; ARRIVE guidelines recommended for reporting animal research, and Sex and Gender in Research

Laboratory animals	Female BALB/c mice (4 weeks old) were purchased from The Jackson Laboratory.<br>BALB/c Tlr5-/- mating pair of mice were a gift of Joon Haeng Rhee, Chonnam National University Medical School, South Korea (Shim JU et al. 2016). BALB/c Tlr5-/- were bred and maintained by the Department of Veterinary Medicine and Surgery of the University of Texas M.D. Anderson Cancer Center.<br><br>Female C57BL/6J (6-9 weeks old) were purchased from The Jackson Laboratory.
Wild animals	Study did not used wild animals.
Reporting on sex	All mice in this study were female.
Field-collected samples	Study did not involve samples collected from the field.
Ethics oversight	The IACUC board is an internal independent review board at University of Texas MD Anderson Cancer Center, and the Iaucuc protocol number if 00001179 for these experiments.<br><br>See the below further statements around oversite.<br><br>Accreditation<br><br>Accreditation and Assurance Documents<br><br>The University of Texas MD Anderson's animal care and use program has been accredited since 1969 by the Association for Assessment and Accreditation of Laboratory Animal Care (AAALAC). The U.S. Department of Agriculture (USDA) inspects the institution for adequate veterinary care. The Office of Laboratory Animal Welfare (OLAW) reviewed and approved the institution's Animal Welfare Assurance submitted in compliance with the Public Health Service (PHS) policy on Humane Care and Use of Laboratory Animals. Related accreditation and assurance documents are provided below.<br><br>USDA Inspection Reports available upon request to Office of Research Oversight & Integrity (OROI). Email IACUC.<br><br>USDA Inspection Report Certificate<br>No. #74-R-0065<br>Last USDA Inspection Date: March 29, 2022<br><br>AAALAC Accreditation<br>No. #000183<br>Last AAALAC site visit March 2022<br><br>PHS Animal Welfare Assurance Statement<br># A3343-01<br>Expires April 30, 2026

Note that full information on the approval of the study protocol must also be provided in the manuscript.

ChIP-seq

Data deposition

☐ Confirm that both raw and final processed data have been deposited in a public database such as GEO.

☐ Confirm that you have deposited or provided access to graph files (e.g. BED files) for the called peaks.

Data access links<br>*May remain private before publication.*	*For "Initial submission" or "Revised version" documents, provide reviewer access links. For your "Final submission" document, provide a link to the deposited data.*
Files in database submission	*Provide a list of all files available in the database submission.*
Genome browser session<br>(e.g. UCSC)	*Provide a link to an anonymized genome browser session for "Initial submission" and "Revised version" documents only, to enable peer review. Write "no longer applicable" for "Final submission" documents.*

Methodology

Replicates	*Describe the experimental replicates, specifying number, type and replicate agreement.*

Sequencing depth	*Describe the sequencing depth for each experiment, providing the total number of reads, uniquely mapped reads, length of reads and whether they were paired- or single-end.*
Antibodies	*Describe the antibodies used for the ChIP-seq experiments; as applicable, provide supplier name, catalog number, clone name, and lot number.*
Peak calling parameters	*Specify the command line program and parameters used for read mapping and peak calling, including the ChIP, control and index files used.*
Data quality	*Describe the methods used to ensure data quality in full detail, including how many peaks are at FDR 5% and above 5-fold enrichment.*
Software	*Describe the software used to collect and analyze the ChIP-seq data. For custom code that has been deposited into a community repository, provide accession details.*

Flow Cytometry

Plots

Confirm that:

☒ The axis labels state the marker and fluorochrome used (e.g. CD4-FITC).

☒ The axis scales are clearly visible. Include numbers along axes only for bottom left plot of group (a 'group' is an analysis of identical markers).

☒ All plots are contour plots with outliers or pseudocolor plots.

☒ A numerical value for number of cells or percentage (with statistics) is provided.

Methodology

Sample preparation	Tumors and spleens were removed with scissors or forceps and weighted. Tumors were chopped into fine pieces and transferred into RPMI media (5% FBS + 10 mM HEPES) containing collagenase (0.2 mg/mL) (Clostridium histolyticum, Sigma) and spleens were suspended in PBS. Tumors were shaken gently at 37°C for 30 minutes prior to tissue disruption. Spleen and tumor tissues were disrupted using a cell strainer (70 µm nylon) (Corning) using a syringe plunge. Nylon mesh was rinsed several times with media. Samples were spin (5 min at 1500 rpm) and re-suspended in 1 mL RBC lysis buffer (Sigma) for 5 minutes and washed 2x with flow buffer (Thermo Fisher). Cells were counted (Nexelom Cellometer), prior to live/dead staining with Zombie UV (Biolegend), incubated for 15 minutes at room temperature and washed 2x with PBS. This was followed by Fc block (CD16/CD32, BD Biosciences), antibody staining and preparation of single-color compensation controls (Ultra Comp eBeads compensation beads, Thermo Fisher). Samples were covered in foil and kept at 4°C overnight. Intracellular staining was performed using Permeabilization Buffer (eBioscience) in conjunction with Foxp3/Transcription Factor Staining Buffer set (eBioscience). Fluorescence minus one (FMO) controls were used where indicated to distinguish between positively and negatively stained cells for FoxP3, Ly6G and Ly6c.
Instrument	Flow cytometry was performed using an LSRII cytometer (Becton Dickinson).
Software	Analysis was performed utilizing FlowJo 10.7.1 (FlowJo, Becton Dickinson).
Cell population abundance	At least 10,000 cells
Gating strategy	Hierarchical gating strategy was employed: First cells were ID with FSC/SSC axis, then live or dead (Zombie Staining), followed by different immunological markers as provided in the Supplementary Information.

☒ Tick this box to confirm that a figure exemplifying the gating strategy is provided in the Supplementary Information.

Magnetic resonance imaging

Experimental design

Design type	*Indicate task or resting state; event-related or block design.*
Design specifications	*Specify the number of blocks, trials or experimental units per session and/or subject, and specify the length of each trial or block (if trials are blocked) and interval between trials.*
Behavioral performance measures	*State number and/or type of variables recorded (e.g. correct button press, response time) and what statistics were used to establish that the subjects were performing the task as expected (e.g. mean, range, and/or standard deviation across subjects).*

Acquisition

Imaging type(s) — *Specify: functional, structural, diffusion, perfusion.*

Field strength — *Specify in Tesla*

Sequence & imaging parameters — *Specify the pulse sequence type (gradient echo, spin echo, etc.), imaging type (EPI, spiral, etc.), field of view, matrix size, slice thickness, orientation and TE/TR/flip angle.*

Area of acquisition — *State whether a whole brain scan was used OR define the area of acquisition, describing how the region was determined.*

Diffusion MRI ☐ Used ☐ Not used

Preprocessing

Preprocessing software — *Provide detail on software version and revision number and on specific parameters (model/functions, brain extraction, segmentation, smoothing kernel size, etc.).*

Normalization — *If data were normalized/standardized, describe the approach(es): specify linear or non-linear and define image types used for transformation OR indicate that data were not normalized and explain rationale for lack of normalization.*

Normalization template — *Describe the template used for normalization/transformation, specifying subject space or group standardized space (e.g. original Talairach, MNI305, ICBM152) OR indicate that the data were not normalized.*

Noise and artifact removal — *Describe your procedure(s) for artifact and structured noise removal, specifying motion parameters, tissue signals and physiological signals (heart rate, respiration).*

Volume censoring — *Define your software and/or method and criteria for volume censoring, and state the extent of such censoring.*

Statistical modeling & inference

Model type and settings — *Specify type (mass univariate, multivariate, RSA, predictive, etc.) and describe essential details of the model at the first and second levels (e.g. fixed, random or mixed effects; drift or auto-correlation).*

Effect(s) tested — *Define precise effect in terms of the task or stimulus conditions instead of psychological concepts and indicate whether ANOVA or factorial designs were used.*

Specify type of analysis: ☐ Whole brain ☐ ROI-based ☐ Both

Statistic type for inference (See Eklund et al. 2016) — *Specify voxel-wise or cluster-wise and report all relevant parameters for cluster-wise methods.*

Correction — *Describe the type of correction and how it is obtained for multiple comparisons (e.g. FWE, FDR, permutation or Monte Carlo).*

Models & analysis

n/a	Involved in the study
☐	☐ Functional and/or effective connectivity
☐	☐ Graph analysis
☐	☐ Multivariate modeling or predictive analysis

Functional and/or effective connectivity — *Report the measures of dependence used and the model details (e.g. Pearson correlation, partial correlation, mutual information).*

Graph analysis — *Report the dependent variable and connectivity measure, specifying weighted graph or binarized graph, subject- or group-level, and the global and/or node summaries used (e.g. clustering coefficient, efficiency, etc.).*

Multivariate modeling and predictive analysis — *Specify independent variables, features extraction and dimension reduction, model, training and evaluation metrics.*
